# Mediation of arsenic mobility by organic matter in mining-impacted sediment from sub‐Arctic lakes: implications for environmental monitoring in a warming climate

**DOI:** 10.1007/s12665-022-10213-2

**Published:** 2022-02-16

**Authors:** Clare B. Miller, Michael B. Parsons, Heather E. Jamieson, Omid H. Ardakani, R. Timothy Patterson, Jennifer M. Galloway

**Affiliations:** 1 Centre for Ore Deposits and Earth Sciences (CODES), Department of Earth Science, University of Tasmania, Hobart, TAS 7001 Australia; 2 Department of Geological Sciences and Geological Engineering, Queen’s University, Kingston, ON K7L 3N6 Canada; 3 Geological Survey of Canada/Commission Géologique du Canada, Natural Resources Canada/Ressources Naturelles Canada, 1 Challenger Drive, Dartmouth, NS B2Y 4A2 Canada; 4 Geological Survey of Canada/Commission Géologique du Canada, Natural Resources Canada/Ressources Naturelles Canada, 3303 - 33 Street N.W., Calgary, AB T2L 2A7 Canada; 5 Ottawa‐Carleton Geoscience Centre, Department of Earth Sciences, Carleton University, Ottawa, ON KIS 5B6 Canada

**Keywords:** Arsenic speciation, Mine waste, Climate change, Contaminant mobility, Environmental monitoring

## Abstract

Arsenic (As) is commonly sequestered at the sediment–water interface (SWI) in mining-impacted lakes through adsorption and/or co-precipitation with authigenic iron (Fe)-(oxy)hydroxides or sulfides. The results of this study demonstrate that the accumulation of organic matter (OM) in near-surface sediments also influences the mobility and fate of As in sub-Arctic lakes. Sediment gravity cores, sediment grab samples, and porewaters were collected from three lakes downstream of the former Tundra gold mine, Northwest Territories, Canada. Analysis of sediment using combined micro-X-ray fluorescence/diffraction, K-edge X-ray Absorption Near-Edge Structure (XANES), and organic petrography shows that As is associated with both aquatic (benthic and planktonic alginate) and terrestrially derived OM (e.g., cutinite, funginite). Most As is hosted by fine-grained Fe-(oxy)hydroxides or sulfide minerals (e.g., goethite, orpiment, lepidocrocite, and mackinawite); however, grain-scale synchrotron-based analysis shows that As is also associated with amorphous OM. Mixed As oxidation states in porewater (median = 62% As (V), 18% As (III); *n* = 20) and sediment (median = 80% As (-I) and (III), 20% As (V); *n* = 9) indicate the presence of variable redox conditions in the near-surface sediment and suggest that post-depositional remobilization of As has occurred. Detailed characterization of As-bearing OM at and below the SWI suggests that OM plays an important role in stabilizing redox-sensitive authigenic minerals and associated As. Based on these findings, it is expected that increased concentrations of labile OM will drive post-depositional surface enrichment of As in mining-impacted lakes and may increase or decrease As flux from sediments to overlying surface waters.

**Supplementary Information:**

The online version contains supplementary material available at 10.1007/s12665-022-10213-2.

## Introduction

Twenty-first century climate warming has disproportionately affected high northern latitudes, causing greater and more rapid increases in temperature and duration of ice-free seasons relative to lower latitudes (ACIA 2005). This warming has promoted increased primary production and transport of terrigenous OM to northern lakes (Frey and McClelland 2009; Prowse et al. 2011; Stern et al. 2012; Griffiths et al. 2017). The enhanced flux of OM influences the biogeochemistry and redox dynamics of near-surface lake sediments (Toevs et al. 2006; McGuire et al. 2009; Rantala et al. 2016; Outridge et al. 2017), and in particular, the mobility of redox-sensitive elements such as As (Martin and Pedersen 2002; Wang and Mulligan 2006). Lakes affected by gold-mining activities commonly have elevated concentrations of As in sediments due to input from tailings, waste rock, effluent, airborne emissions, windblown dusts, and/or mineralized bedrock (DeSisto et al. 2011; Craw and Bowell 2014; Galloway et al. 2015, 2018; Miller et al. 2019; Palmer et al. 2019). Changing redox conditions in the water column and near-surface sediments driven by seasonal variations and longer term changes associated with increased OM flux can cause the release of As from sediments to overlying surface waters (Martin and Pedersen 2002; Bauer and Blodau 2006; Couture and Van Cappellen 2011; Anawar et al. 2013; Barrett et al. 2019; Palmer et al. 2019; Schuh et al. 2019). The influence of dissolved OM (DOM) and changing redox conditions on the geochemical cycling of As is well documented (e.g., Redman et al. 2002; Bauer and Blodau 2006; Mladenov et al. 2015; Lawson et al. 2016). However, the specific role of various organic fractions which comprise sediment OM (i.e., amino acids, chlorophyll, phytoplankton, benthic microalgae, cutinite, and fulvic acid; Simon et al. 2002) are not as well understood (Langner et al. 2011; Anawar et al. 2013; Biswas et al. 2019), especially in lakes (Galloway et al. 2018). As climate warming continues to impact sensitive sub-Arctic and Arctic ecosystems, an improved understanding of the effects of increasing OM on the long-term stability of As is needed to predict the impacts of future climate variations on As mobility in northern lake environments.

The post-depositional mobility of As in lacustrine sediment is governed by the solid-phase speciation of As, which depends primarily on the redox conditions of the sediment and associated porewaters (Smedley and Kinniburgh 2002). Under oxic conditions, As is sequestered through adsorption and co-precipitation with authigenic and detrital Fe- and manganese (Mn)-(oxy)hydroxides at the SWI (Dixit and Hering 2003). The onset of reducing conditions, through burial in sediments or increased abundance of labile OM that consumes oxygen (O_2_) upon decay, allows for the sequestration of dissolved As through sorption and co-precipitation reactions involving authigenic sulfides (Farquhar and Livens 2002; Bostick et al. 2005; Lowers et al. 2007; Le Pape et al. 2017; Schuh et al. 2019). The development of reducing conditions is progressive with depth, and results in an oxic-to-anoxic transition zone in the upper sediment column, in the hypolimnion of seasonally stratified lakes or, in some cases, under prolonged ice cover (e.g., Palmer et al. 2019). At this redox interface, decreasing concentrations of available O_2_ result in the release of As through reductive dissolution of Fe- and Mn-(oxy)hydroxides (Wolthers et al. 2005; Bennett et al. 2012; An et al. 2017). Under changing redox conditions, the formation of organo-mineral aggregates (OMAs) and/or Fe monosulfides (FeS; e.g., mackinawite) may promote the sequestration of metal(loid)s (e.g., As, Cu, Cd, Ni, Pb, and Zn) through co-precipitation with, or sorption to, these authigenic phases (Coles et al. 2000; Farquhar et al. 2002; Gallegos et al. 2007; Du et al. 2018; Zhou et al. 2021). However, the effectiveness of these mechanisms in sequestering elements from bottom waters to sediments in natural environments is difficult to predict (Moon and Peacock 2012; Chen et al. 2014; Kleber et al. 2015; An et al. 2017; Vega et al. 2017; Qu et al. 2019).

There is a growing consensus that specific fractions of sediment OM play important roles in the mobility of metal(loid)s, such as As, in low-temperature environmental systems (e.g., peat bogs, deltaic sediments, soils, and lakes) (Simon et al. 2002; Langner et al. 2011; Galloway et al. 2018; Wang et al. 2018; Biswas et al. 2019). In these environments, As binding to OM may occur via ternary complexes through the formation of a metal-cation bridge (i.e*.,* Fe (III), Al (III), Ca (II)) (Ritter et al. 2006; Sharma et al. 2010; Hoffmann et al. 2013), thiol-bonding to sulfhydryl groups of OM (Langner et al. 2011; Wang et al. 2018), or complexation with the oxygen-containing functional groups of OM (Tessier et al. 1996; Biswas et al. 2019). The role of sediment OM in the sequestration of As may be of particular importance under transitional redox conditions where Fe reduction and S oxidation occur simultaneously and As is susceptible to remobilization. Characterization of both the mineralogical and biogeochemical processes that regulate the mobility of As in mining-impacted, freshwater aquatic systems is necessary to inform the monitoring and remediation of these environments.

Arsenic concentrations in many lakes surrounding former gold mine sites in northern Canada are elevated above Canadian Council of Ministers of the Environment (CCME) guidelines and regional background due to historical mining activities (Wagemann et al. 1978; Bright et al. 1994, 1996; Groves et al. 1998; Andrade et al. 2010; Galloway et al. 2012, 2015, 2018; Palmer et al. 2015, 2019, 2021; Jamieson et al. 2017; Schuh et al. 2018, 2019; Miller et al. 2019). Previous work (Galloway et al. 2018; Miller et al. 2019, 2020) shows that labile OM affects As mobility and fate through its influence on the redox environment of near-surface sediment and long-term stability of As-bearing minerals. However, the grain-scale associations between As and sediment OM have not been studied in detail and are important for determining the mechanism of control on As mobility. This study aims to address the knowledge gaps presented in Galloway et al. (2018) and Miller et al. (2019, 2020) and focuses specifically on determination of the origin of sedimentary OM in sub-Arctic lakes, and the variable influence of aquatic- and terrigenous-derived OM on the mobility of As in three mining-impacted lakes located in the central Northwest Territories (NT), Canada.

## Study area and previous work

**Fig. 1:**
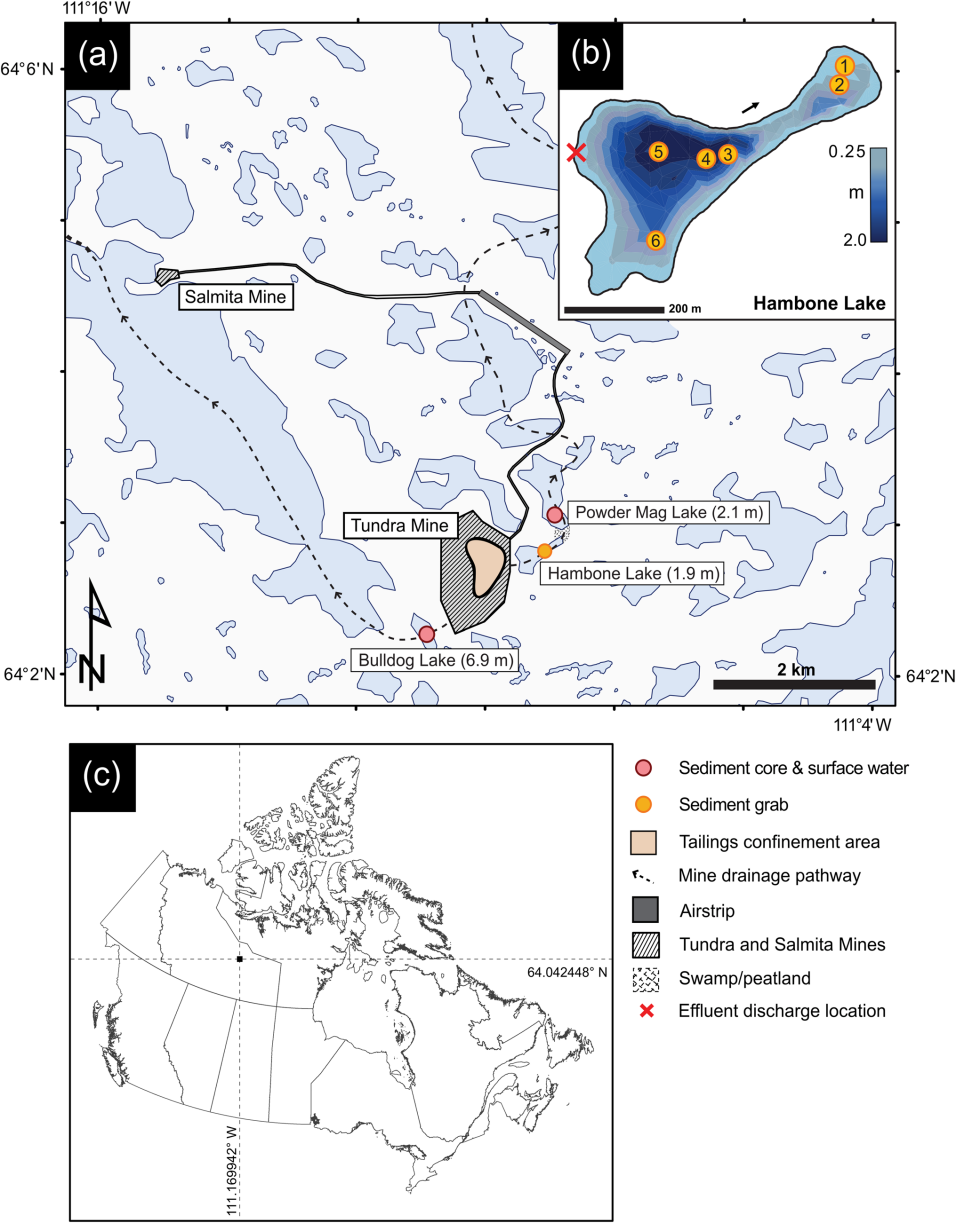
Map showing **a** sampling locations and predominant mine drainage pathways originating from the former Tundra Mine site (dashed lines), and maximum lake depths (in parentheses) figure after Miller et al. (2019); **b** bathymetry map of Hambone Lake with numbered locations of sediment grab samples (modified from Miller et al. 2019); **c** study site located at the intersection of graticules

The study area is the former Tundra Mine, which is located in the central portion of the Slave Geological Province of the Canadian Shield and is 80 km north of the present-day tree line (Fig. 1). The area is part of the transition zone between discontinuous and continuous permafrost with numerous small lakes and organic deposits occurring in low-lying areas (Seabridge Gold Inc., 2010; Karunaratne, 2011; AECOM, 2015). Small lakes, such as those investigated in this study, may be ice-covered from mid-October to late June (Palmer et al. 2019).

The former Tundra and Salmita mines are approximately 240 km NE of Yellowknife, NT (Fig. 1). Approximately, 285,000 oz of gold produced at grades ranging from 18.4 to 27.8 g·t^−1^ were produced during two phases of mining, from 1964 to 1968 and 1983 to 1987 (Ransom and Robb 1986; Silke 2009). Ore was processed at the Tundra Mine facilities and wastes were deposited into an adjacent lake (location of Tailings Confinement Area (TCA); Fig. 1). During the second period of mining, tailings-core dams were built to reinforce this lake and create a tailings confinement area (Fig. 1). The Tundra Mine site has been in care and maintenance since 1999 under the stewardship of the Contaminants and Remediation Directorate (CARD) of Crown-Indigenous Relations and Northern Affairs Canada (CIRNAC). At the beginning of remediation activities in 2007, approximately 155,000 m^3^ of waste rock, 240,000 m^3^ of tailings, and 1,280,000 m^3^ of impacted water were present on-site (URS 2005; Golder Associates Ltd. 2008). In lakes surrounding the tailings impoundment at the Tundra Mine site, As concentrations in surface water range from 0.80 to 105 µg·L^−1^ and from 24 to 1,010 mg·kg^−1^ in near-surface sediments (Staples, 2014; Miller et al. 2019). Arsenic concentrations in the near-surface sediment of all five lakes sampled by the authors in 2016 exceed the CCME Interim Sediment Quality Guideline (ISQG; 5.9 mg·kg^−1^) and Probable Effect Level (17 mg·kg^−1^; CCME 2001a, b). The disposal of waste rock, overtopping and seepage of tailings ponds, weathering and airborne deposition of waste rock and tailings, discharge of treated tailings effluent, and natural weathering of mineralized bedrock have all contributed to the elevated As concentrations in lakes around the former Tundra Mine (Miller et al. 2019). The site is currently in an adaptive management and long-term monitoring phase, following the completion of remediation activities in August 2018 (AECOM 2018). The oxidation of mining-derived sulfides (e.g., arsenopyrite) and authigenic As-bearing framboidal pyrite are the predominant sources of increased porewater As concentrations in the near-surface sediment of Bulldog Lake. Conversely, in Hambone and Powder Mag lakes, the seepage and overflow of tailings waters and disposal of treated effluent contributed As, in dissolved or suspended form, to the near-surface sediment. Despite the oxic nature of the overlying surface waters of Bulldog (DO = 7.80 mg·L^−1^) and Powder Mag (DO = 3.60 mg·L^−1^) lakes, the presence of mixed As oxidation states in both the aqueous and solid-phase suggests that the contaminant source and sediment redistribution processes have influenced the post-depositional mobility of As and associated redox species (Fe, S) at the SWI despite oxygenated conditions at mid-water column depths (Martin and Pedersen 2002; Miller et al. 2019; Schuh et al. 2019). Due to their shallow nature, it is unlikely these lakes are stratified (Fig. 1).

Sediment cores were collected from Powder Mag Lake (64.05114° N, 111.15042° W) and Bulldog Lake (64.03758° N, 111.18337° W) on the Tundra Mine property in March 2016. Sediment grab samples were collected from Hambone Lake (64.047033° N, 111.154958° W) in July 2016 (Fig. 1). Mean sedimentation rates in the near-surface sediment of Powder Mag (52 year·cm^−1^) and Bulldog (40 year·cm^−1^) lakes are higher than average for regional lakes (70 year·cm^−1^; Crann et al. 2015) and this increased sedimentation rate is attributed to mining activities at Tundra and Salmita mines and associated landscape disturbance (Miller et al. 2019).

## Methods and materials

### Sample collection and preparation

Sediment cores were collected from the frozen surface of Powder Mag and Bulldog lakes using a gravity corer with polycarbonate core tubes (7.5 × 60 cm). All sediment cores were collected from the deepest location in each lake to sample the zone of accumulation (Blais and Kalff 1995). Immediately following collection, cores were vertically extruded and sub-sampled at 1-cm resolution in a glove bag filled with high purity (99.998%) nitrogen (N_2_) in the field. Sediment was sub-sampled into N_2_–flushed 50–mL polypropylene centrifuge tubes (Corning® Falcon®) and refrigerated prior to shipment to the Geological Survey of Canada–Atlantic (GSC–A) in Dartmouth, Nova Scotia, for porewater extraction. Near-surface (0–15 cm) sediment grab samples were collected from Hambone Lake using an Ekman dredge sampler and homogenized prior to analysis. These sediment samples were frozen and sent to Queen’s University, Kingston, Ontario, for sub-sampling and preparation. Aliquots of sediment were freeze-dried at 1.0 Pa and − 50 °C for organic geochemistry and petrography (0.5 g) and inorganic geochemical analyses (0.5 g). A separate aliquot was dried at room temperature in a glove bag filled with high purity N_2_ to preserve solid-phase As speciation (Huang and Ilgen, 2006). This N_2_-dried material was used for scanning electron microscope (SEM)-based automated mineralogy, electron microprobe analysis (EPMA), synchrotron-based bulk XANES, micro-X-ray fluorescence (μ-XRF), and micro-X-ray diffraction (μ-XRD) to determine the mineralogy of As-bearing hosts and As speciation in the sediments of lakes sampled near Tundra Mine.

Porewater was extracted from the sediment core sub-samples by centrifugation (Thermo Scientific Sorvall Legend™ XF Centrifuge). Samples were prepared and handled following the protocol detailed in Miller et al. (2019) for filtered cations, filtered anions, total As, and total inorganic As speciation. For each sample, pH was measured with a hand-held pH meter (HACH H138 MiniLab ISFET). Due to low water content, Eh could not be measured.

### Sediment and porewater geochemistry

Metal(loid) concentrations in porewaters were measured at the Inorganic Geochemical Research Laboratory of the Geological Survey of Canada in Ottawa. Analyses of major elements were performed by Inductively Coupled Plasma–Atomic Emission Spectroscopy (ICP-AES) using a Perkin-Elmer 3000 DV. Trace elements were analyzed using Inductively Coupled Plasma–Mass Spectroscopy (ICP-MS) with a Thermo Corporation X-7 Series II. Relative standard deviation (RSD) ranges from 0.08 to 1.40% and mean percent difference (MPD) ranges from 0.12 to 1.98% for As in laboratory duplicates. Unfiltered field duplicates demonstrate higher RSD and MPD (2.44% and 3.45%, respectively) than filtered duplicates (2.05% and 2.90%). Arsenic was below detection limit (0.1 µg·L^−1^) in all field blanks (*n* = 4).

The relative proportions of dissolved As (V), As (III), and a residual fraction (As_R_) in water samples were determined by hydride generation–atomic fluorescence spectrometry (HG-AFS; model PSA 10.055 Millennium Excalibur) at the Université de Montréal. The residual fraction likely includes As species that are not detected using HF-AFS, including thioarsenates in sulfidic waters and non-reducible organoarsenic compounds (Planer-Friedrich and Wallschläger 2009; PS Analytical 2018).

Element concentrations in sediment samples (*n* = 211) were determined by ICP-MS (1F/AQ250 package) following digestion by a modified *aqua regia* treatment (1:1:1 HCl:HNO_3_:H_2_O at 95 °C) at Acme Analytical Laboratories (Bureau Veritas), Vancouver, British Columbia. Partial digestion with *aqua regia* was used as key elements of interest (i.e., As and S) may be lost through volatilization in more aggressive, near-total digestions (Parsons et al. 2012, 2019). In blind duplicate samples, relative standard deviation (RSD) ranged from 0.21 to 7.22% and mean percent difference (MPD) ranged from 0.29 to 10.21% for As. A certified stream sediment reference material (STSD-3; Lynch 1990) was included with each sample set. The mean measured As concentration in STSD-3 was 24.34 ± 0.81 mg·kg^−1^ (*n* = 8) vs. an expected concentration of 22 ± 6 mg·kg^−1^ for As following *aqua regia* digestion (RSD 3.3%). Mean sulfur concentration (0.15 ± 0.01%; *n* = 8) was within the standard deviation of the certified value (0.14 ± 0.04%).

### Sediment organic matter characterization

The amount and type of OM in sediments (Hambone Lake *n* = 6; Powder Mag Lake *n* = 30; Bulldog Lake *n* = 37) was determined by Rock–Eval 6® programmed pyrolysis at the Geological Survey of Canada, Calgary (Lafargue et al. 1998). In duplicate samples, RSD ranges from 0 to 9% and MPD ranges from 0 to 13% for the organic constituents of interest (S1, S2, S3, total organic carbon (TOC); Sanei et al. 2005). Further details regarding sample preparation, methodologies, and analytical precision are provided in Supplementary Information SM1. Particle size analysis for near-surface sediment is presented in Miller et al. (2019).

The TOC in sediment represents the sum of all organic compounds. In recent sediments, the S1 fraction of OM is comprised of aquatic-derived OM (e.g., algal-derived lipids; amino acids, chlorophyll, small volatile molecules, a fraction of humic material, and proteins and other macromolecules) and S2 compounds are derived from the biomacromolecule structure of algal cell walls and other aquatic biological matter, such as phytoplankton and copepods (Sanei et al. 2005; Carrie et al. 2012). Terrestrial plant materials (i.e., conifer needles, roots, and bark), in addition to humic and fulvic acids, comprise the S3 fraction (Carrie et al. 2012; Albrecht et al. 2015).

The distribution and origin of sediment OM was assessed using polished, 1-cm epoxy mounts (*n* = 5) and qualitative petrographic analysis following methods outlined by Reyes et al. (2006). Incident white light and fluorescent light microscopy were conducted using a Zeiss Axioimager II microscope system (50 × magnification) equipped with the Diskus-Fossil system. Fluorescence microscopy was conducted using ultraviolet G 365 nm excitation with a 420 nm filter. Organic matter was characterized based on the classification of macerals in International Committee for Coal Petrology (1971), Sanei et al. (2005), and Reyes et al. (2006).

Comparison of optical properties (using fluorescence spectrometry) between samples that had and had not previously been examined using SEM and EPMA analysis suggests that exposure to an electron beam resulted in thermal alteration of the OM. Similar alteration of OM in lithified rocks by ion milling was demonstrated by Sanei and Ardakani (2016), but to the best of our knowledge, the effects of prolonged electron beam exposure on OM in unconsolidated sediment have not been investigated. As a result, SEM/EPMA analysis and organic petrography were conducted on different sub-samples from the same sediment interval.

### Solid-phase As speciation analysis

Eleven N_2_-dried lake sediment samples (Hambone Lake *n* = 3; Powder Mag Lake *n* = 4; Bulldog Lake *n* = 4) were selected for detailed As speciation analysis based on bulk elemental composition and position within each core. Polished thin sections (35 to 50 μm thickness) were prepared at Vancouver Petrographics following specifications by Schuch et al. 2018.

#### SEM-based automated mineralogy and electron microprobe analyses

The relative distribution of As-hosting solid phases in selected sediment samples was completed through automated mineralogy, using a FEI™ Quanta 650 Field Emission Gun Environmental SEM, and Mineral Liberation Analyzer software. This integrated technique quantifies mineral phases through a combination of backscatter electron (BSE) image analysis and energy-dispersive X-ray spectroscopy (EDS) (Buckwalter-Davis 2013). Miller et al. (2019) describe the operating conditions, the mineral reference library used for phase classification, and the calculations used to quantify the relative contribution of each As-hosting phase. Sparse phase liberation (SPL) analysis mode with a user-defined BSE greyscale range (120 to 255) was used to selectively identify As-hosting phases but does not provide bulk mineralogy information (Fandrich et al. 2007). Fine-grained aggregates of poorly crystalline, As-bearing Fe-sulfides and Fe-(oxy)hydroxides (FeS_x_/FeO), identified by Miller et al. (2019), co-occur with OM and were targeted for analysis using a JEOL JXA-8230 electron microprobe operating in wavelength-dispersive (WDS) mode. The small size of these grains and uneven topography of the polished surface can cause X-ray scattering and inaccurate peak intensity (Rönnhult et al. 1987), therefore, the EPMA results are considered semi-quantitative.

#### Synchrotron-based µ-XRF and µ-XRD

Arsenic-bearing grains associated with sediment OM were identified through combined SEM-based automated mineralogy, EPMA, and fluorescent light microscopy analysis. These grains were subsequently targeted for synchrotron-based μ-XRF and μ-XRD analysis using micro-focused X-rays at beamline 13-ID-E at the Advanced Photon Source (APS), Argonne National Laboratory (Chicago, IL). Due to the penetrative nature of synchrotron-based analysis, thin sections were used for synchrotron-based analysis, instead of 1-cm epoxy pucks, to prevent the diffraction of X-rays from mineral phases deeper in the sample, in addition to the target grains. X-ray fluorescence maps and XRD patterns were collected using an incident beam energy of 18.0 keV and a 2 μm beam diameter. Two-dimensional, continuous µ-XRF/XRD mapping was completed at frame rates of 50 to 100 ms per pixel. Specific points identified on μ-XRF maps were subsequently selected as targets for longer duration μ-XRD measurements. The fluorescent radiation was measured using a Hitachi 4-element Vortex ME4 silicon drift diode positioned at 90° to the incident beam and connected to a Xspress 3 digital X-ray multi-channel analyzer system. X-ray diffraction was measured using a Perkin-Elmer XRD1621 digital flat panel detector placed 400 mm from the sample and operating in transmission mode. Post hoc processing and analysis of µ-XRF maps was performed using Larch software (Version 0.9.46; Newville 2019). Micro-XRF element maps included strontium (Sr) to identify and delineate detrital minerals (e.g., feldspars, micas, and clay minerals) within the sediment. Dioptas software (Prescher and Prakapenka 2015) was used to calibrate and integrate XRD data; phase identification was performed in X’Pert HighScore Plus.

#### Bulk X-ray absorption near-edge structure (XANES)

To determine the relative distribution of As oxidation states in the sediments, As K-edge XANES data were collected at Sector 20-BM of the APS. Preparation of samples for these analyses (Hambone Lake *n* = 3; Powder Mag Lake *n* = 3; Bulldog Lake *n* = 3) followed procedures described by Van Den Berghe et al. (2018). Standard materials were selected to cover the principal oxidation states exhibited by As in lacustrine systems (− 1 to + 5; Supplementary Information Table ST1). Operational conditions and post hoc data processing are detailed in Miller et al. (2020). The distribution of oxidation states reported are component sums, normalized to 100%.

## Results

### Sediment and porewater geochemistry

**Fig. 2:**
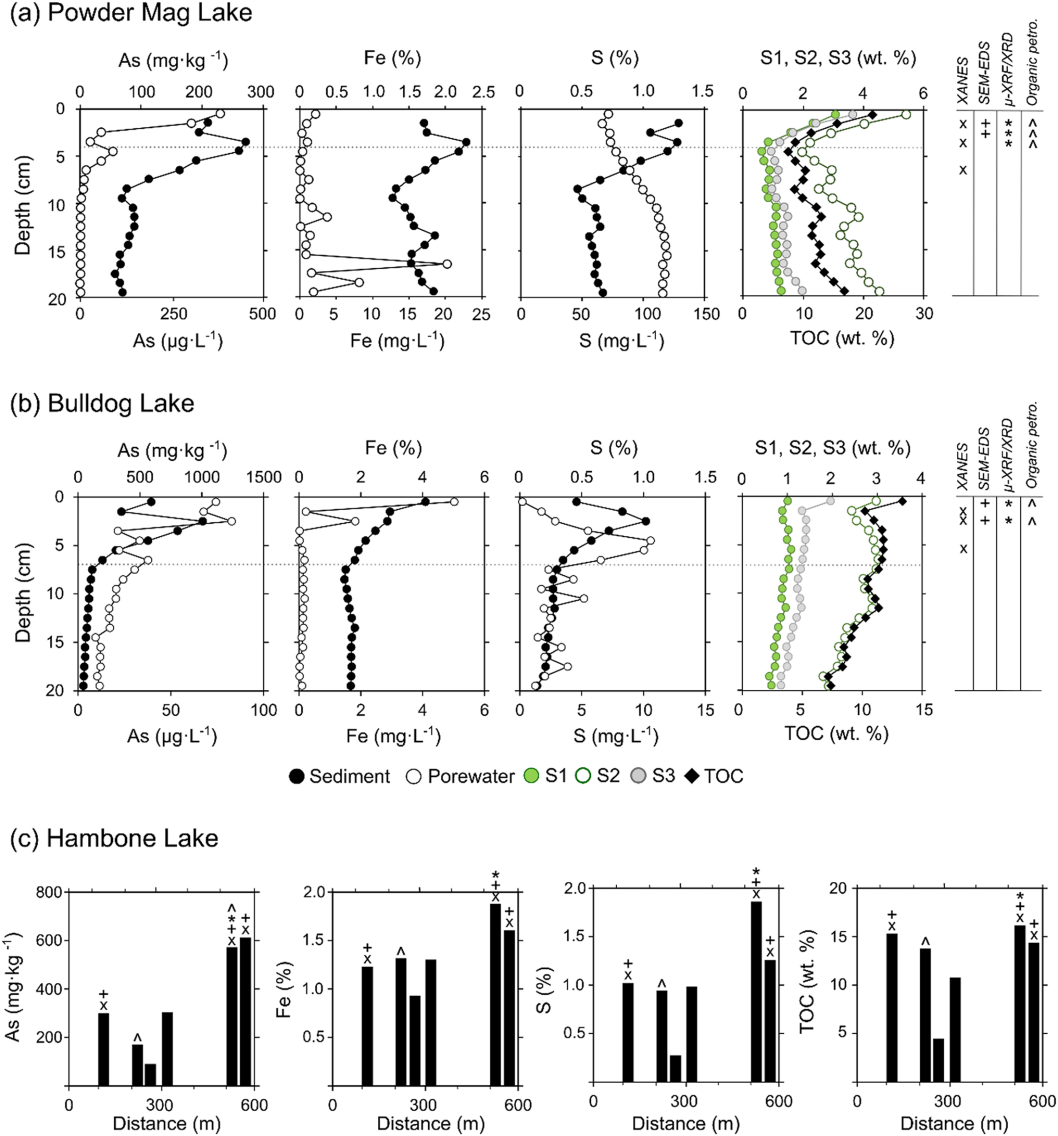
Downcore plots of As, Fe, and S concentrations in sediment and porewater, and sediment organic matter composition in the near-surface sediment of **a** Powder Mag Lake and **b** Bulldog Lake. Note the scale of the x-axes varies between each lake. The horizontal dotted line represents the onset of mining activities in 1964 (modified from Miller et al. 2019). **c** Arsenic, Fe, S and TOC concentrations in surface sediment (0–10 cm) of Hambone Lake with distance from effluent discharge (organic matter fractions (S1, S2, S3) available in Supplementary Information). Micro-analytical techniques used to characterize selected samples are shown on each plot using various symbols (legend shown on right in **a** and **b**)

Near-surface sediment As concentrations range from 80 to 1,010 mg·kg^−1^ in the three lakes sampled near Tundra Mine (Fig. 2). The highest solid-phase As concentrations were observed in Bulldog Lake (median = 570 mg·kg^−1^; range = 200 to 1010 mg·kg^−1^; *n* = 7) with maximum values occurring from 2 to 3 cm in the 37 cm deep sediment profile. Maximum dissolved As concentrations in porewater were observed in Powder Mag Lake (maximum: 383 μg·L^−1^) and are an order of magnitude higher than those measured in Bulldog Lake (maximum 83 μg·L^−1^). The concentration and down-core distribution of redox-sensitive elements (Fe and S) varies between the sediment cores collected from Bulldog and Powder Mag lakes (Fig. 2; Supplementary Information Tables ST2, ST3). Based on grab samples, near-surface sediment concentrations of As in Hambone Lake ranged from 79.6 to 622 mg·kg^−1^ (*n* = 6; Supplementary Information ST4).

Downcore profiles of porewater and sediment concentrations in Powder Mag and Bulldog lakes range demonstrate that dissolved As concentrations range from 21 to 383 μg·L^−1^ in the near-surface sediment (Fig. 2; Supplementary Information Tables ST5, ST6). In these two lakes, maximum dissolved As concentrations in sediment occur at or just below the sediment–water interface (SWI; Fig. 2). In Powder Mag Lake, maximum porewater As concentrations occur at the SWI, while in Bulldog Lake peak concentrations are observed at 2.5 cm depth.

Concentration profiles of selected dissolved redox-sensitive species (Fe, S) are shown in Fig. 2. In Bulldog Lake, porewater distribution of dissolved Fe is characterized by a sharp subsurface concentration gradient in the shallow porewaters with the maximum concentration (5.02 mg·L^−1^) occurring at the SWI. Conversely, from 0 to 5 cm, dissolved S concentrations increased non-linearly and reach a subsurface maximum at 3.5 cm depth. Profiles of dissolved Fe and S are notably different in Powder Mag Lake with minimal surface enrichment observed.

The pH of porewaters in the near-surface sediment of Bulldog and Powder Mag lakes is circum-neutral, ranging from 5.7 to 7.5 (median = 5.8; *n* = 11; Supplementary Information ST5). The highest pH values are observed in Powder Mag Lake (range = 6.3–7.5; Supplementary Information ST6). Porewater data are not available for Hambone Lake and surface waters were not collected over the duration of this study; however, past environmental monitoring recorded surface water pH from 6.4 to 7.95 (AANDC 2013; Golder Associates Ltd. 2016). This range is similar to circum-neutral surface water pH values measured by the authors in 2016 for Powder Mag (pH = 7.01) and Bulldog lakes (pH = 6.75) (Miller et al. 2019).

### Sediment organic matter characterization

Total organic carbon (TOC) content in the near-surface sediment of all lakes studied ranges from 8.7 to 21.5 wt. % (median = 11.4 wt. %; *n* = 25), with maximum concentrations observed at the SWI in Powder Mag Lake (Fig. 2; Supplementary Information Tables ST2, ST3, ST4). In both Powder Mag and Bulldog lake sediment cores, the highest OM concentrations are observed at the SWI. The majority of labile OM (pyrolysable carbon) is composed of S2 (median = 2.4 wt. %; range = 1.2–5.4 wt. %; *n* = 25). In Hambone Lake, the highest TOC (15.5 wt. %) and S2 (4.96 wt. %) occur at the site farthest from the treated tailings effluent discharge location (HAM-2; Fig. 1).

**Fig. 3:**
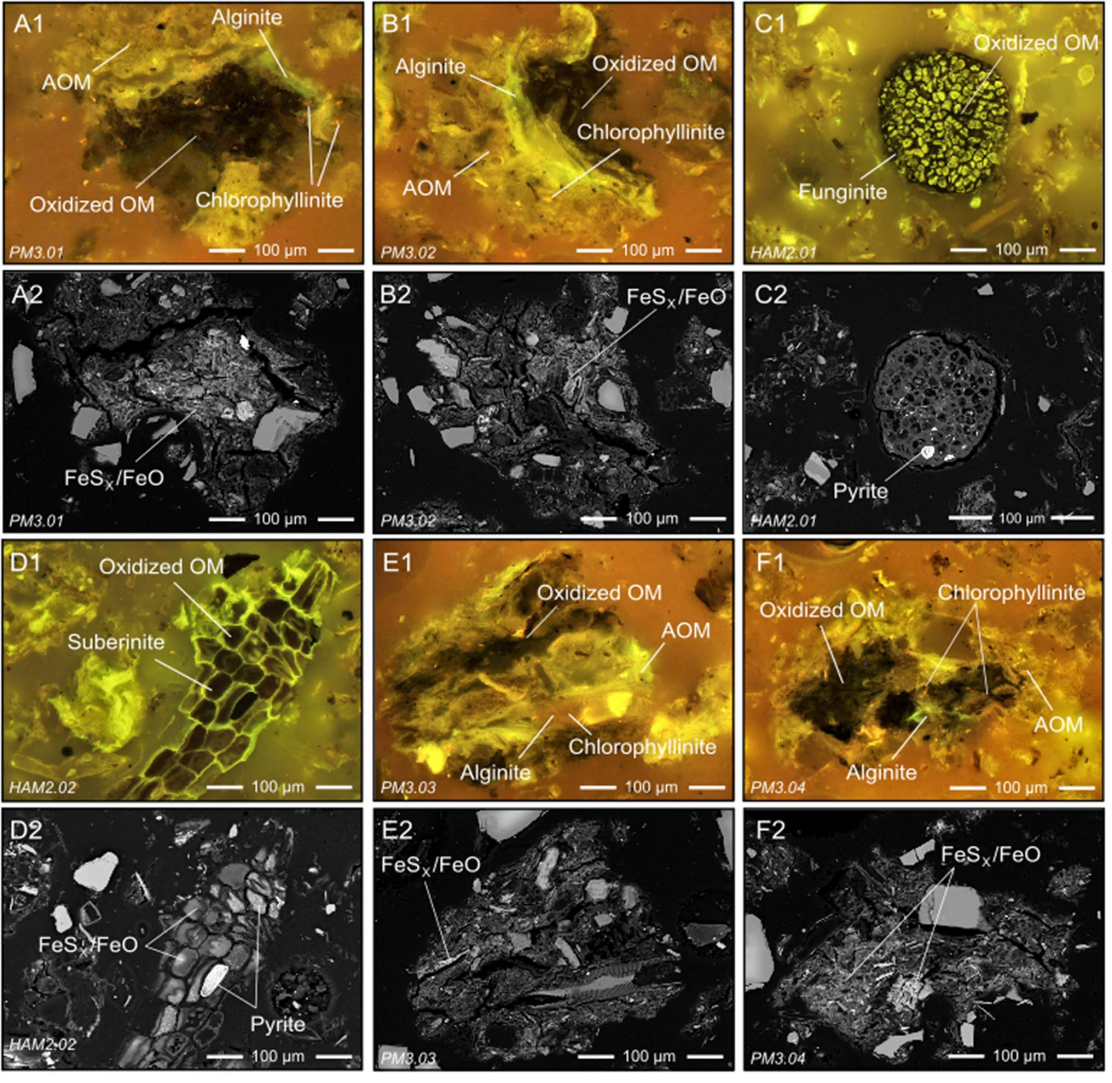
Paired (1) fluorescence-light photomicrographs under oil immersion and (2) SEM-BSE images of organo-mineral aggregates (**A**, **B**, **E**, **F**) and terrigenous OM (**C**, **D**) preserved in near-surface sediment of Powder Mag and Hambone lakes. Organic macerals include suberinite (**D1**; green fluorescing), funginite (**C1**; green to yellow fluorescing inner layer), chlorophyllinite (**A1**, **B1**, **E1**, **F1**; pink to red fluorescing), oxidized OM (**A1**, **B1**, **C1**, **D1**, **E1**, **F1**; brown fluorescing), alginate (A1, B1. F1; green fluorescing), and amorphous OM (AOM) (**A1**, **B1**, **E1**, **F1**; green to yellow fluorescing). **A** = Powder Mag (grain PM3.01; 2–3 cm); **B** = Powder Mag (grain PM3.02; 2–3 cm); **C** = Hambone Lake (grain HAM2.01; Sample 2); **D** = Hambone Lake (grain HAM2.02; Sample 2); **E** = Powder Mag (grain PM3.03; 2–3 cm); **F** = Powder Mag (grain PM3.04; 2–3 cm)

Figure 3 juxtaposes fluorescence-light photomicrographs and SEM-BSE imagery of OMAs (A, B, E, and F) and terrigenous OM (C, D) preserved in near-surface sediment of Powder Mag and Hambone lakes. Combining these analytical techniques demonstrates the heterogeneous nature of these particles and allows for the comparative and qualitative analyses of both the inorganic and organic components. Classification and identification of sediment OM, based on optical properties (fluorescence and reflectance) and morphology indicate that OM in the near-surface sediment of lakes in the Tundra Mine region is derived from both aquatic and terrestrial sources. Woody fragments and resin, suberin (i.e., roots and bark; Fig. 3D), cutinite (i.e., cuticle of leaves and stems) and funginite (Fig. 3C) from terrestrial vegetation are common in the near-surface sediment. Aquatic-derived OM is also abundant and comprised primarily of alginate (benthic and planktonic; Fig. 3). Chlorophyllinite is derived from chlorophyll pigments and can be either aquatic or terrestrial in origin (Fig. 3E1). Organo-mineral aggregates (comprised of amorphous OM, particulate macerals (e.g., alginate and chlorophyllinite), oxidized amorphous OM (Fig. 3A1), and mineral matter) are abundant in all sediment samples studied (Fig. 3). Oxidation of amorphous OM and terrigenous OM is commonly observed and results in a spectral shift towards lower energy fluorescence wavelengths (i.e., redshift; Fig. 3). SEM-BSE imagery and reflected fluorescence-light microscopy show that sulfide and Fe-(oxide) mineral precipitates are closely associated with oxidized aquatic and terrestrial-derived OM (Fig. 3). Unoxidized aquatic and terrestrial-derived OM is identifiable in the sediment as it does not display a spectral shift and preserves its characteristic fluorescence (Fig. 3).

### Solid and aqueous As speciation

#### SEM-based automated mineralogy and electron microprobe analyses

**Fig. 4:**
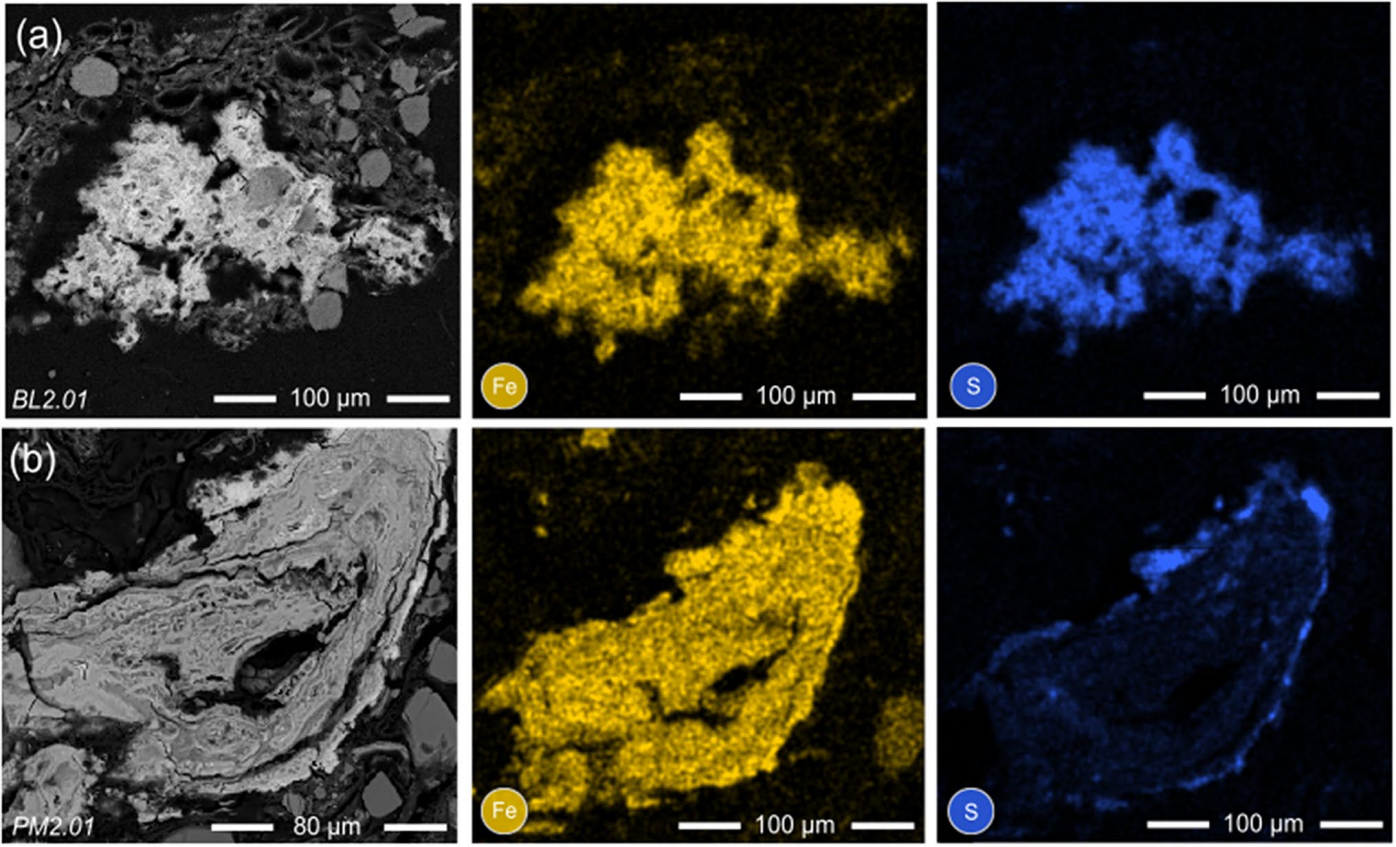
SEM-BSE images and EDS element maps of **a** OMAs and **b** terrigenous OM preserved in the near-surface sediment of **a** Bulldog Lake (grain BL2.01; 1–2 cm) and **b** Powder Mag Lake (grain PM2.01; 1–2 cm)

Fine-grained aggregates of Fe-sulfides and Fe-(oxy)hydroxides (FeS_x_/FeO) are abundant in the near-surface sediment of all lakes studied and observed in association with both OMAs and terrigenous OM. SEM-based EDS mapping illustrates the precipitation of both Fe and S within these authigenic minerals (Fig. 4). Arsenic concentration associated with these authigenic minerals ranges from less than detection to 2.0 wt% (48 particles; 89 spots); however, the heterogeneous nature and fine grain size suggest the EMPA results should be considered semi-quantitative.

#### Synchrotron-based µ-XRF and µ-XRD

**Fig. 5:**
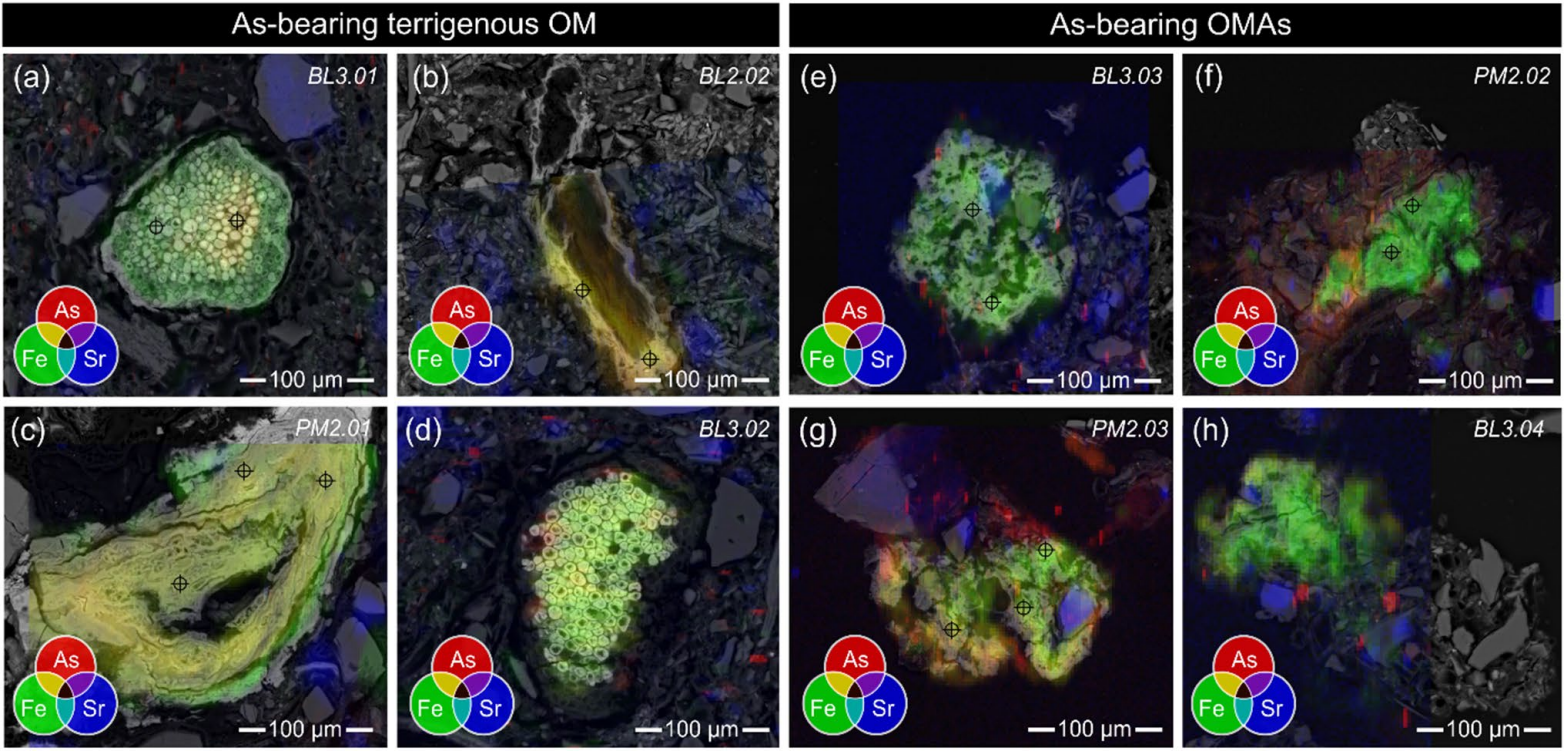
Micro-XRF element maps showing As-Kα (red), Fe-Kα (green), and Sr-Kα (blue) intensities overlain on SEM-BSE images of As-bearing terrigenous OM (**a**, **b**, **c**, **d**) and OMAs (**e**, **f**, **g**, **h**). Color intensities are not comparable between maps and are not intended to represent exact concentrations. Cross hairs demonstrate location of EPMA analysis, note that symbol is larger than beam diameter. A) Bulldog (grain BL3.01; 2–3 cm); B) Bulldog (grain BL2.02; 1–2 cm); C) Powder Mag (PM2.01; 1–2 cm); D) Bulldog (BL3.02; 2–3 cm); E) Bulldog (grain BL3.03; 2–3 cm); F) Powder Mag (PM2.02; 1–2 cm); G) Powder Mag (PM2.03; 1–2 cm); H = Bulldog (grain BL3.04; 2–3 cm)

**Fig. 6:**
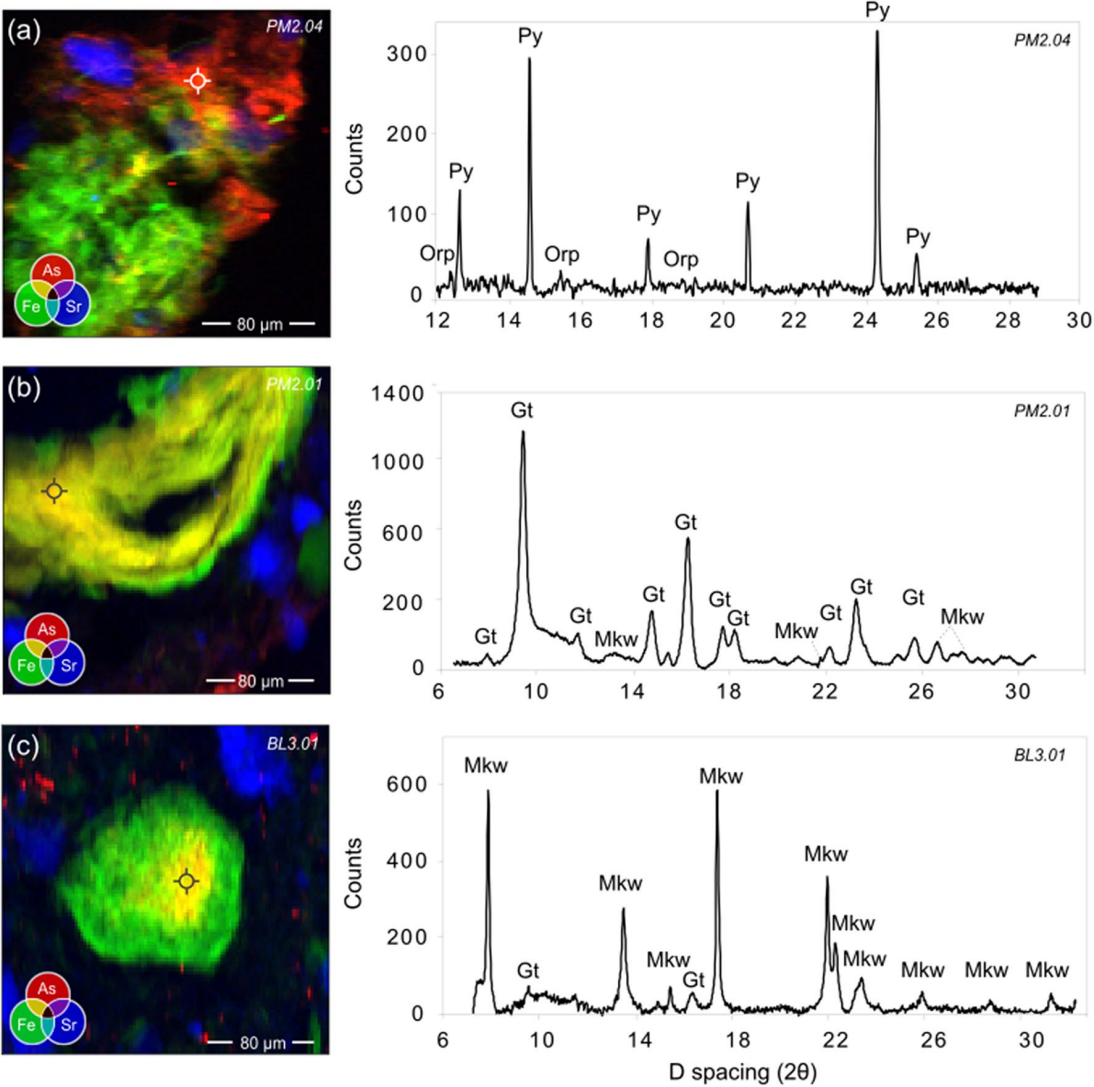
Micro-XRF element maps showing As-Kα (red), Fe-Kα (green), and Sr-Kα (blue) intensities of As-bearing OMAs (**a**) and terrigenous OM (**b**, **c**). Color intensities are not comparable between maps and are not intended to represent exact concentrations. Integration of the µ-XRD patterns shows the presence of discrete grains of pyrite (Py) and orpiment (Orp) associated with OMAs, which are comprised of a heterogenous mixture of detrital (not shown) and authigenic minerals (e.g., feroxyhyte (Feroxy)). Authigenic minerals associated with terrigenous OM are comprised of goethite (Gt) and mackinawite (Mkw). Cross hairs demonstrate location of µ-XRD analysis, note that symbol is larger than beam diameter. A = Powder Mag Lake (grain PM2.05; 1–2 cm); B = Powder Mag (PM2.01; 1–2 cm); C = Bulldog (BUL3.01; 2–3 cm). Additional analyses are provided in Supplementary Information

Micro-XRF results confirm both the heterogeneous nature of OMAs and terrigenous OM, as well as the association of As with both types of OM (Fig. 5). Organo-mineral aggregates are comprised of both fine-grained detrital grains (i.e., silicates, phyllosilicates, quartz, and hematite) and mineral phases that may be authigenic in origin (i.e., ferrihydrite, feroxyhyte, goethite, maghemite, mackinawite, and pyrite; Fig. 6; Supplementary Information Figure SF2). Terrigenous OM is associated with goethite, ferrihydrite, maghemite, lepidocrocite, pyrite, and mackinawite (Fig. 6).

Synchrotron-based micro-XRF element maps demonstrate that As is physically associated with terrigenous OM and OMAs (Fig. 5). Arsenic is generally observed with authigenic FeS_x_/FeO minerals and is dispersed throughout the Fe-bearing inorganic components associated with terrigenous OM (Fig. 5a–d). In contrast, As in OMAs is instead more commonly observed either as micron-sized grains within the organic material or dispersed throughout the amorphous OM (Fig. 5e–h). Micro-XRD analyses of the OMAs suggest these micron-sized grains may be As-bearing sulfide minerals, such as orpiment (As_2_S_3;_ Fig. 6a); however, µ-XRD analysis results did not provide identifiable diffraction patterns for many of these As-rich regions (Fig. 6).

#### HG-AFS and bulk As XANES

**Fig. 7:**
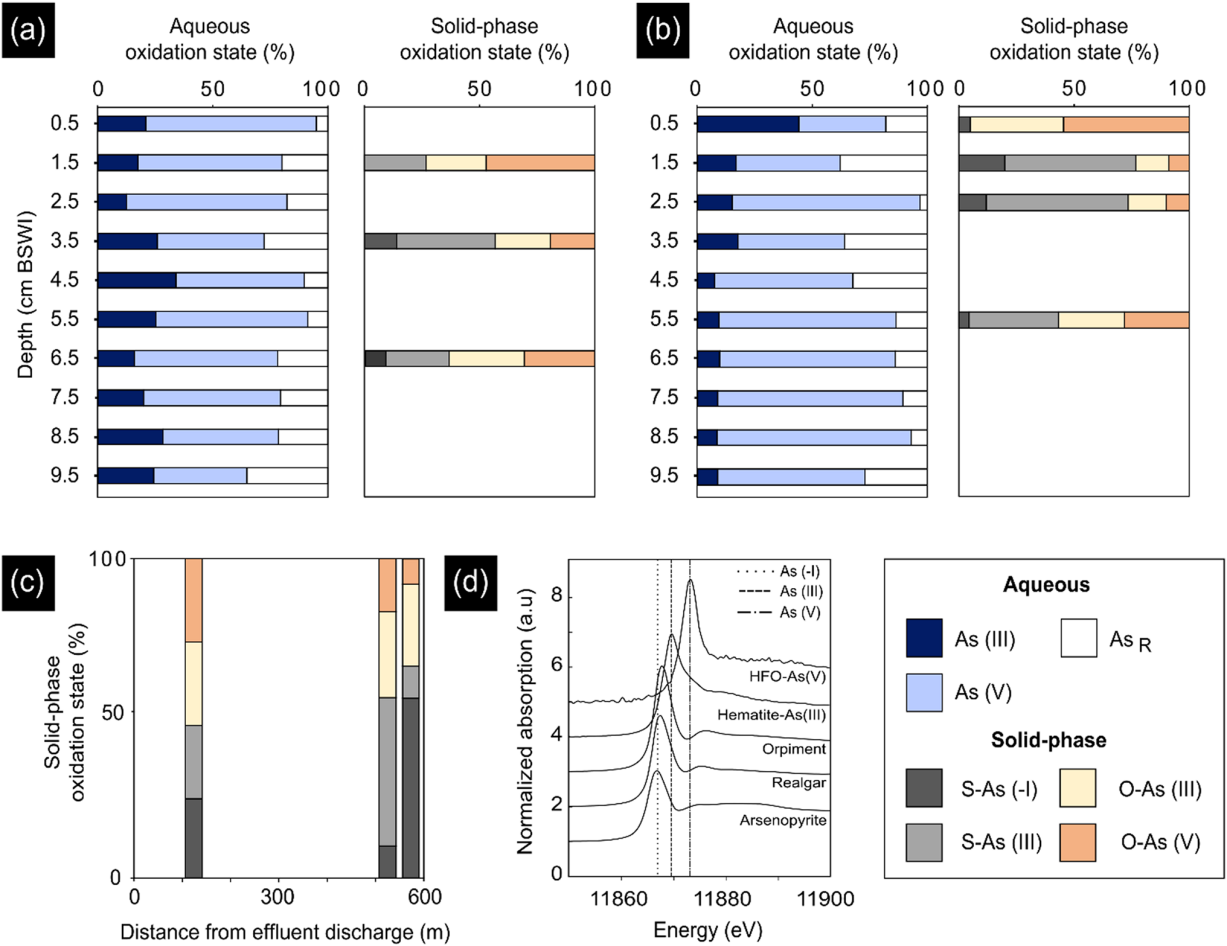
Relative distribution of As species in porewater (HG-AFS) and sediment (bulk XANES) with depth in the **a** Powder Mag Lake and **b** Bulldog Lake sediment cores and **c** Hambone Lake with distance from the effluent discharge location in surface sediments (Fig. 1). **d** XANES spectra of standards. Averaged XANES spectra and LCF fitting for samples is provided in Supplementary Information

In near-surface (0–10 cm) sediment porewaters of Powder Mag and Bulldog lakes, As (V) is the predominant inorganic aqueous As species, except at the SWI (0–1 cm) in Bulldog Lake, where As (III) is the dominant oxidation state (Fig. 7). Maximum dissolved As concentrations in both lakes (Fig. 2) occur where As (V) proportions are highest and proportions of As_R_ are lowest (Fig. 7). In Bulldog Lake, a trend of increasing proportions of As (V) with depth is observed, while below a depth of 1 cm, the relative proportion of As (III) is consistently around 10% of total porewater As concentration. In Powder Mag Lake, no consistent down-core trend in the proportions of both As (III) and As_R_ is observed in near-surface porewaters.

The As K-edge XANES spectra demonstrate that reduced solid-phase As species (As (-I), As (III)) predominate in the near-surface sediment of all lakes studied, accounting for an average of 80% (*n* = 9) of total As (Fig. 7; Supplementary Information Table ST7). In Powder Mag Lake, the relative proportions of O-bound As (III) and As (V) species (11,869.7 eV and 11,873.2 eV, respectively) are higher than S-coordinated As (-I) or As (III) (11,867.0 and 11,867.7 eV, respectively). At the SWI, O-bound As (III) and As (V) account for 25% and 47% of total As, respectively. An increase in S-bound As species is observed at 3.5 cm depth and corresponds to maximum As concentrations (271 mg kg^−1^) in the near-surface sediment (Fig. 2A). Conversely, in Bulldog Lake, S-coordinated As species account for approximately 40% of solid-phase As species in the upper 3 cm of sediment. A trend of decreasing S-coordinated As species and increasing O-bound As species is observed with depth in Bulldog Lake. In Hambone Lake, ratios of O- and S-bound As species are similar in sediment close to the treated tailings effluent discharge location; however, the relative proportions of S-bound species increase with distance toward the peatland at the NE end of the lake that may be a source of S (Figs. 1, 7C).

## Discussion

### Sources of As and organic matter to lakes

The primary mechanisms of inorganic As delivery to the near-surface sediment of Hambone, Powder Mag, and Bulldog lakes are related to legacy gold-mining practices with a minor contribution from the weathering of mineralized bedrock (Miller et al. 2019). Arsenic may also be introduced to the lake sediments from the watershed in association with aquatic and terrestrial-derived OM. Dissolved As may bioaccumulate in aquatic organisms living in the water column, such as phytoplankton and zooplankton, to concentrations orders of magnitude higher than those in ambient waters (Eisler, 1988; Hellweger et al. 2003; Lopez et al. 2017). Abundant labile OM is observed both petrographically (alginate) and geochemically (S1 and S2) in the near-surface sediment of Bulldog and Powder Mag lakes, suggesting that aquatic organisms are accumulating As and delivering it to near-surface sediment (Fig. 2; Caumette et al. 2012). Previous studies have shown that algal scavenging is an important mechanism for transporting Hg to the bottom sediments in high Arctic lakes (Outridge et al. 2007, 2017; Stern et al. 2012); however, the importance of this process is less certain in sub-Arctic lakes with more diverse sources of both autochthonous and allochthonous OM. In surface waters, As may form aqueous and colloidal complexes with OM and dissolved or suspended Fe (III) and eventually accumulate in the near-surface sediment (Ritter et al. 2006; Sundman et al. 2014). The formation of these organo-ferric complexes and their affinity for binding trace metals is influenced by seasonal fluctuations in surface water physicochemistry (i.e., temperature, redox conditions, and light penetration) and microbiological metabolism (Shirokova et al. 2013; Palmer et al. 2019).

High dissolved organic carbon (DOC) and total Fe in both Powder Mag (DOC = 16.6 mg·L^−1^; Fe = 0.39 mg·L^−1^) and Hambone (DOC = 16 mg·L^−1^; Fe = 0.55 mg·L^−1^) surface waters, reported by INAC (2005) and Miller et al. (2019), suggest that the formation of complexes between arsenate, suspended Fe-(oxy)hydroxide colloids, and OM, play a significant role in the transport of both As and OM to the bottom sediments (Ritter et al. 2006; Golder Associates Ltd., 2016). In addition, petrographic evidence of terrigenous OM (i.e., cutinite, funginite, and sporinite) and the presence of detrital As-bearing minerals demonstrate that the watershed is contributing both OM and As to these lake systems (Fig. 3).

### OMAs and As sequestration

Organo-mineral aggregates, comprised of amorphous OM, particulate macerals (e.g., sporinite and alginate), and both authigenic and detrital minerals, are common in the near-surface sediment of the lakes studied near Tundra Mine (Fig. 4). In these aggregates, mixtures of detrital minerals (i.e., silicates, quartz, and pyrrhotite) and fine-grained authigenic mineral phases (i.e., ferrihydrite, goethite, maghemite, mackinawite, and pyrite) occur together with amorphous OM (Fig. 3). Arsenic is associated with these aggregates as: (1) discrete clusters, (2) evenly dispersed within the amorphous OM matrix, and (3) sorbed to and/or co-precipitated with authigenic FeS_x_/FeO portions of the grain (Figs. 5, 6).

Arsenic-bearing OMAs are present at and below the SWI in both Bulldog and Powder Mag lakes where redox conditions progressively change from oxic to anoxic. This transition in redox conditions is evident in the aqueous speciation of As and porewater profiles of dissolved Fe, As, and S concentrations. A shift in aqueous As speciation from mainly As (V) in overlying waters (Powder Mag Lake: 78% As (V); Bulldog Lake: 95% As (V)) to increasing proportions of As (III) in the near-surface sediment occurs in both lakes (Miller et al. 2019; Fig. 7). Elevated concentrations of dissolved Fe and As in the shallow porewaters and increasing concentrations of dissolved S with depth in the sediment of both lakes (Fig. 2) also indicates the onset of reductive processes in the near-surface sediments, as described in the study by Martin and Pedersen (2002). Solid-phase speciation and mineralogy also provides evidence of the progressive onset of reducing conditions, with a shift from O-bound As (V) species at the SWI to S-coordinated As (-I) or As (III) at ~ 3 cm in both the lakes (Fig. 7).

Within these OMAs, As most commonly occurs as discrete clusters (Fig. 5). Micro-XRD suggests these are small grains of orpiment (Fig. 6). The presence of orpiment is supported by bulk XANES, which indicates that S-coordinated As (III) accounts for approximately 60% of solid-phase As in the near-surface sediment of Bulldog Lake. A shift in fluorescence color (from green to red wavelengths) is observed in the amorphous OM associated with these aggregates. This fluorescence shift is caused by the oxidation of OM as it acts as an electron donor to drive sulfide mineralization, suggesting that these discrete As minerals are forming in situ (Fig. 3; Davis et al. 1990) and that OM is promoting the formation of As-bearing sulfide minerals, possibly as a substrate for microbial growth (Galloway et al. 2018). These grain-scale observations suggest that redox conditions are likely to be heterogeneous at a given depth in the near-surface sediments, and will depend in part of the spatial distribution of particulate OM. The role of reactive OM in the precipitation of authigenic As-bearing minerals has previously been demonstrated in both laboratory (e.g., Kirk et al. 2004, 2010; Liu et al. 2017; Zhou et al. 2021) and field-based sediment studies (e.g., Langner et al. 2011; Galloway et al. 2018; Wang et al. 2018; Miller et al. 2020).

Within OMAs, coupled µ-XRF and fluorescence microscopy analyses demonstrate that As is dispersed throughout the amorphous OM (Fig. 5). These regions of elevated As concentrations did not diffract, suggesting that As may be bound to OM through the formation of ternary complexes with Fe ions and/or bonding with hydroxyl groups (Caumette et al. 2012; Biswas et al. 2019). The results of this study suggest that O-bound As in the near-surface sediment of Bulldog, Hambone, and Powder Mag lakes is attributed to sorption to and/or co-precipitation with Fe-(oxy)hydroxides. In addition, this study demonstrates that the association of As with OMAs may contribute to the abundance of O-bound As species, particularly below the SWI where Fe-(oxy)hydroxides would begin to reductively dissolve (Fig. 7).

Less commonly, As is observed co-precipitated with or sorbed to authigenic Fe-(oxy)hydroxide and Fe-sulfide minerals within the OMAs (Fig. 5). Regions of authigenic mineralization with higher proportions of As are most commonly comprised of a mixture of ferrihydrite and goethite (Fig. 6). At mildly acidic to circum-neutral pH, As (III) and As (V) have similar affinities for Fe-(oxy)hydroxides, suggesting that As is associated with these phases through sorption. This may explain the presence of both As valence states in the near-surface sediment of all three lakes studied (Fig. 7; Dixit and Hering 2003). Mackinawite mineralization is also observed within these OMAs and, based on µ-XRF maps, trace amount of As have been observed to co-precipitate or sorb to this phase (Fig. 5). However, As is more commonly observed as fine-grained As-sulfides. These observations support the laboratory-based studies conducted by Wolthers et al. (2005) who observed the formation of poorly crystalline As_2_S_3_ precipitates at the surface of FeS minerals at neutral pH.

### Arsenic sequestration with terrigenous OM

Arsenic-bearing terrigenous OM is observed in the near-surface sediment of all lakes studied. Ranging in size from 20 to 200 µm, these organic macerals are primarily derived from terrestrial vegetation (i.e., roots, leaves, and spores; Figs. 3, 4). The modern landscape of the Tundra Mine region consists of tundra shrubland with stunted spruce and fir, and organic-rich depressions, indicating terrestrial weathering is a source of OM to these lakes (Ecosystem Classification Group 2008, 2012). The organic structure of these macerals contains mixtures of authigenic mineral precipitates, including goethite, ferrihydrite, maghemite, lepidocrocite, pyrite, and mackinawite (Fig. 6; Supplementary Information Figure SF1). The heterogenous nature of these grains, comprised of both sulfide and oxide precipitates, suggests that they formed at an oxic-to-anoxic transition zone in the sediment column.

The association between organic carbon in sediments and reactive Fe phases has been found to play a significant role in the stabilization of OM under dynamic redox conditions. These recent studies suggest that the interactions between Fe and organic compounds, such as those derived from vascular plants, stabilize both authigenic Fe minerals and OM as conditions become moderately reducing at redox interfaces (Lalonde et al. 2012; Riedel et al. 2013; Chen et al. 2015). This has important implications for shallow lake sediments where both authigenic Fe minerals and terrigenous OM act as potential sorbents and influence the mobility of As and other elements.

Combined µ-XRD and EPMA analyses demonstrate that authigenic Fe-(oxy)hydroxide and/or Fe-sulfide minerals associated with terrigenous OM commonly contain more than 1 wt. % As (Fig. 4). Micro-XRF analysis shows that As is concentrated at the center of these grains and decreases outwards; however, within a terrigenous OM maceral, no consistent patterns were observed in either the distribution of authigenic minerals or the association of As with these phases (Fig. 5). Recent research investigating the immobilization of metal(loid)s under transitional redox conditions suggests that the timing of authigenic mineral (e.g., FeS_x_) precipitation influences the effectiveness of this process in contaminant removal via sequestration in sediments (Vega et al. 2017; Du et al. 2018). For example, scavenging of dissolved As is more effective in systems where As co-precipitates with FeS in comparison to in settings where FeS forms in the sediment prior to introduction of the contaminant (Vega et al. 2017). The high As concentrations observed in Fe-sulfide minerals associated with terrigenous OM in lakes near Tundra Mine suggest that these sulfides precipitated authigenically in the mining-impacted lake sediments, sequestering dissolved As during their formation. In addition, the co-precipitation of OM with Fe-(oxy)hydroxides can alter their affinity for metal sorption at mid- to low pH (Du et al. 2018). The results of this study suggest that while the influx of terrigenous OM is likely to result in As release from minerals to pore waters by enhancing reductive dissolution, it may also reduce the flux of dissolved As to overlying surface waters by facilitating the precipitation of authigenic Fe-(oxy)hydroxide and FeS_x_ minerals under changing redox conditions.

### Implications for long-term environmental monitoring

The results of this study help to improve knowledge about the long-term fate of As in lake systems when increased autochthonous OM production and terrigenous (i.e., allochthonous) OM delivery promotes a shift from oxidizing to reducing conditions in the near-surface sediments and overlying waters. The degradation of accumulated labile OM will increase sediment oxygen demand, driving the progressive onset of reducing conditions and influence the stability of redox-sensitive elements (e.g., Fe, S, and As) and minerals (e.g., Fe-oxides and sulfides). Changing redox conditions and shallowing of the sediment redoxcline are expected to release As to pore waters and overlying surface waters via reductive dissolution of Fe-(oxy)hydroxides or the oxidation of sulfides (e.g., Martin and Pedersen 2002; Galloway et al. 2018; Schuh et al. 2019). However, this study demonstrates that sediment OM may also promote the sequestration of As at this transitional redox boundary by facilitating the formation of fine-grained As-sulfides, the precipitation of authigenic As-bearing FeS_x_/FeO minerals, and the direct sorption of As to amorphous OM functional groups. As a result, the diffusion of As into porewaters may be abated by the presence of sediment OM in near-surface sediment. The net effect of these competing processes is difficult to predict as the present-day mineral hosts for As vary between lakes, which, in turn, will influence their long-term stability under changing redox conditions (Miller et al. 2019, 2020; Palmer et al. 2019).

Based on the results of this study, it is expected that increased concentrations of aquatic- and terrestrially derived labile OM will drive the redistribution of As in shallow lake sediments resulting in surface enrichment of As. This will pose a challenge for interpretation of long-term monitoring data and may make it difficult to distinguish between mining impacts and the influence of current warming in the sub-Arctic. It is important that the influence of natural, climate-driven biogeochemical processes on the post-depositional mobility of As is considered within monitoring programs aiming to delineate potential mining impacts.

## Limitations and future work

Sorption and co-precipitation mechanisms are controlled by the biogeochemical conditions of near-surface lake sediments. Differences in the total metal(loid) concentrations, pH, TOC, and OM composition of Hambone, Powder Mag, and Bulldog lake sediments will influence the effectiveness of sediment OM in the sequestration of As. The limited sample size of this study does not allow for broad predictions about how other sub-Arctic lakes might behave. This investigation does, however, demonstrate that sediment OM plays an important role in the cycling of As in near-surface sediment, and highlights the need for further study to determine the influence of seasonality, intra- and inter-lake variances, and reaction mechanisms between As (III), As (V) and sediment OM. While combining organic petrography techniques (Rock Eval, fluorescence microscopy) with geochemical and mineralogical speciation methods (SEM-based automated mineralogy, EPMA, synchrotron-based μ-XRF/XRD) revealed novel observations, the sample preparation for each of these methods limits the ability of sequential analysis by different techniques on individual grains. For example, 1-cm epoxy pucks are preferred to thin sections for fluorescence microscopy as the transmission of reflected light through a thin section may interfere with the OM fluorescence spectra. Conversely, for µ-XRD, thin sections are preferred to minimize the number of particles diffracting during analysis. In addition, this study suggests that prolonged electron beam exposure on OM in unconsolidated sediment may result in thermal alteration and influence fluorescence microscopy interpretations. As a result, optimization of a method that allows grains to be analyzed sequentially would provide further evidence for the relationship between sediment OM, authigenic minerals, and the cycling of elements of concern.

## Conclusions

The association between As and sediment OM was examined to determine the influence of increased flux of aquatic- and terrigenous-derived OM on the long-term mobility of As in three mining-impacted lakes of the Tundra Mine region. Detailed geochemical and organic petrological analyses suggest that increased primary production in lakes and weathering of terrestrial vegetation, associated with climate warming and changing biogeochemistry of high northern latitude environments, is impacting the mobility of legacy contaminants. While increased loading of labile OM will change redox conditions in lake sediments, driving the release of As to overlying surface waters via reductive dissolution of As-bearing Fe-(oxy)hydroxides, sediment OM may also provide an additional substrate for As sequestration and increase retention of As in sediments, reducing its concentrations in the water column.

Increased aquatic-derived OM, interpreted to be a result of seasonal fluctuations and climate warming, was observed in the near-surface sediment of all lakes studied. Clumping and/or binding together of mineral matter and aquatic-derived OM formed OMAs, comprised of amorphous OM, particulate macerals (e.g., sporinite and alginate) and both authigenic and detrital mineral matter. Arsenic is associated with these phases, primarily in the form of authigenic, fine-grained, poorly crystalline, As-sulfides (e.g., orpiment). Within these OMAs, As is also associated with amorphous OM and, to a lesser extent, with authigenic FeS_x_/FeO precipitates (i.e., pyrite, goethite, ferrihydrite, and mackinawite).

Increased input of terrigenous OM to the near-surface sediment of these mining-impacted lakes may facilitate the precipitation of reactive authigenic FeS_x_/FeO minerals, (e.g., lepidocrocite, ferrihydrite, goethite, mackinawite, and pyrite) providing a substrate for As sequestration. The presence of these As-bearing substrates at and below the SWI suggests that terrigenous-derived OM in lake sediments plays a role in stabilizing redox-sensitive authigenic minerals (sulfides and Fe-(oxy)hydroxides), and associated As, as redox conditions progressively change from oxic to anoxic.

Through combined microscale geochemical and OM speciation analysis, this study demonstrates that under dynamic redox conditions increased algal and terrestrially derived OM will mediate the flux of As to overlying surface water by: (1) facilitating the precipitation of authigenic sulfides, (2) enhancing the stability of authigenic oxides, and/or (3) allowing for the direct sequestration of As to OM. These findings provide strong evidence that sediment OM plays an important role in the sequestration of As under changing redox conditions and results demonstrate that changes in sediment redox conditions as a result of climate warming will affect the mobility and fate of As in northern lakes. This study improves knowledge about As mobility in lake sediments and can help to improve long-term monitoring strategies for As and other trace metal(loid)s (e.g., Cd, Pb, Sb, Zn) that are affected by redox conditions. Based on the results of this study, it is expected that increased concentrations of aquatic and terrigenous OM will result in enrichment of As in near-surface lake sediments, making it more challenging to differentiate mining impacts from those associated with current and forecasted warming trends in the sub-Arctic.

## Supplementary Information

Below is the link to the electronic supplementary material.

Supplementary file1 (DOCX 618 kb)

## Data Availability

The primary data associated with this manuscript have been included as Supplementary Information. Supporting data and further information on methods and materials are part of a Ph.D. thesis by the lead author and are available at http://hdl.handle.net/1974/27588.
