# Supplementary material for: Mediation of arsenic mobility by organic matter in mining-impacted sediment from sub‐Arctic lakes: implications for environmental monitoring in a warming climate
Source: Environ Earth Sci. 2022 Feb 16;81(4):137. doi: 10.1007/s12665-022-10213-2 (PMC8850223; doi:10.1007/s12665-022-10213-2)

**Supplementary Information**

*Article Title: Mediation of arsenic mobility by organic matter in mining-impacted sediment from sub‐Arctic lakes: Implications for environmental monitoring in a warming climate*

Clare B. Miller^a,b*^, Michael B. Parsons, Heather E. Jamieson, Omid H. Ardakani, R. Timothy Patterson, Jennifer M. Galloway

^a^ Centre for Ore Deposit and Earth Sciences (CODES), University of Tasmania, Hobart TAS 7001, Australia.

^b^ Department of Geological Sciences and Geological Engineering, Queen's University, Kingston, ON, K7L 3N6, Canada

* Corresponding author. Tel +61 3 6226 2425 *E-mail address:* clare.miller@utas.edu.au (C.B. Miller)

***Methods SM1:*** *Detailed methodology for programmed pyrolysis (Rock-Eval 6 ®; Vinci Technologies, France).*

Sediment sub-samples (Hambone Lake *n* = 6; Powder Mag Lake *n* = 30; Bulldog Lake *n* = 37) were first subjected to pyrolysis under an inert atmosphere (N_2_). During pyrolysis, the quantity of free/volatile hydrocarbons present in the sample (S1, mg HC·g^-1^) and the amount of hydrocarbon released by the thermal cracking of OM (S2, mg HC·g^-1^) were detected with a Flame Ionization Detector. Simultaneously, S3CO_2_ (mg CO_2_·g^-1^) and S3CO (mg CO·g^-1^), derived from thermal cracking of oxygen-bearing compounds, were measured by infrared spectroscopy (Lafargue et al., 1998; Carrie et al., 2012). Following the pyrolysis stage, the sample was transferred to an oxidation oven and all remaining OM (S4CO_2_, mg CO_2_·g^-1^; S4CO, mg CO·g^-1^) and residual carbon (RC, %) was measured. Total organic carbon (TOC; wt %), was determined from the sum of all carbon released during pyrolysis and oxidation (TOC = pyrolysable organic carbon (PC %) + residual organic carbon (RC%)). The hydrogen index (HI = S2 × 100/TOC, mg HC/g TOC) and oxygen index (OI = S3 × 100/TOC, mg CO_2_·g^-1^ TOC) were calculated. Organic matter fractions S1, S2, and S3 were converted to weight % values by multiplying by 0.083 (Sanei and Goodarzi, 2006).

***Table ST1:*** *Model compounds for post-hoc Linear Combination Fitting XANES analysis*

|  | Formula | As Species | Source | Edge Position | As wt. %* |
| --- | --- | --- | --- | --- | --- |
| Arsenopyrite | FeAsS | As(-I)-S | Deloro, ON, Canada | 11,867.0 | 46.01 |
| Realgar | As_4_S_4_ | As(II)-S | Hunan, China | 11,867.5 | 70.03 |
| Orpiment | As_2_S_3_ | As(III)-S | Moldawa, Hungary | 11,867.7 | 60.90 |
| Hematite-As(III) | 0.026As·Fe_2_O_3_^A^ | Fe_2_O_3_-As(III) | A | 11869.7 | 1.21* |
| HFO-As(V) | 0.0012As·Fe(OH)_3_^A^ | Fe(OH)_3_-As(V) | A, B | 11873.2 | 0.08* |

^*^ Determined by ICP-MS; ^A^ Adsorption procedures by Liu et al. (2006); ^B^ Synthesized following Schwertmann & Cornell (2000) and Liu et al. (2006)

**Table ST2:** Concentrations of As, S, Fe, Mn and organic matter fractions (S1, S2, S3, TOC) in the Powder Mag Lake (POW) sediment core (64.05114° N, 111.15042° W).

| **Sample** | **Depth** | **As** | **S** | **Fe** | **Mn** | **S1** | **S2** | **S3** | **TOC** |
| --- | --- | --- | --- | --- | --- | --- | --- | --- | --- |
| *Units* | *cm* | *mg·kg^-1^* | *wt. %* | *wt. %* | *mg·kg^-1^* | *mg HC/g^A^* | *mg HC/g^A^* | *mg HC/g^A^* | *wt. %* |
| *Detection Limit* | *--* | *0.1* | *0.02* | *0.01* | *1.0* | *0.01* | *0.01* | *0.01* | *--* |
| POW 0-1 | 0.5 | *IS^B^* | *IS* | *IS* | *IS* | 37.0 | 65.1 | 44.0 | 21.5 |
| POW 1-2 | 1.5 | 210 | 1.29 | 1.71 | 102 | 28.2 | 48.6 | 29.3 | 15.6 |
| POW 2-3 | 2.5 | 195 | 1.06 | 1.75 | 97.0 | 19.0 | 35.3 | 20.1 | 11.3 |
| POW 3-4 | 3.5 | 271 | 1.28 | 2.29 | 108 | 10.1 | 26.6 | 14.7 | 8.67 |
| POW 4-5 | 4.5 | 260 | 1.20 | 2.19 | 112 | 7.57 | 23.7 | 11.4 | 7.46 |
| POW 5-6 | 5.5 | 191 | 0.98 | 1.86 | 106 | 8.54 | 28.5 | 12.7 | 8.69 |
| POW 6-7 | 6.5 | 163 | 0.84 | 1.73 | 110 | 10.7 | 35.6 | 14.3 | 10.3 |
| POW 7-8 | 7.5 | 114 | 0.65 | 1.50 | 102 | 10.5 | 35.0 | 13.8 | 9.97 |
| POW 8-9 | 8.5 | 77.2 | 0.46 | 1.33 | 96.0 | 9.26 | 30.3 | 11.7 | 8.58 |
| POW 9-10 | 9.5 | 69.5 | 0.50 | 1.28 | 95.0 | 10.3 | 35.9 | 13.3 | 9.88 |
| POW 10-11 | 10.5 | 87.4 | 0.61 | 1.45 | 111 | 13.3 | 43.3 | 16.5 | 12.1 |
| POW 11-12 | 11.5 | 89.7 | 0.62 | 1.52 | 120 | 13.3 | 46.0 | 17.8 | 13.0 |
| POW 12-13 | 12.5 | 89.0 | 0.65 | 1.57 | 116 | 12.2 | 40.3 | 16.1 | 11.6 |
| POW 13-14 | 13.5 | 81.2 | 0.56 | 1.86 | 109 | 12.7 | 38.9 | 16.0 | 11.4 |
| POW 14-15 | 14.5 | 79.7 | 0.58 | 1.72 | 112 | 13.4 | 44.2 | 17.5 | 12.7 |
| POW 15-16 | 15.5 | 66.0 | 0.60 | 1.54 | 114 | 13.8 | 45.6 | 17.5 | 12.9 |
| POW 16-17 | 16.5 | 66.6 | 0.62 | 1.53 | 122 | 13.0 | 42.6 | 16.3 | 12.0 |
| POW 17-18 | 17.5 | 58.0 | 0.60 | 1.64 | 123 | 13.6 | 47.5 | 18.1 | 13.4 |
| POW 18-19 | 18.5 | 66.0 | 0.63 | 1.68 | 126 | 14.4 | 50.1 | 21.3 | 15.1 |
| POW 19-20 | 19.5 | 70.7 | 0.67 | 1.84 | 134 | 15.3 | 54.7 | 23.8 | 16.8 |
| POW 20-21 | 20.5 | 68.9 | 0.67 | 1.95 | 150 | 16.7 | 62.2 | 27.2 | 19.1 |
| POW 21-22 | 21.5 | 52.6 | 0.51 | 1.78 | 148 | 13.4 | 47.3 | 20.7 | 14.7 |
| POW 22-23 | 22.5 | 59.7 | 0.63 | 1.91 | 158 | 13.2 | 52.6 | 25.7 | 17.5 |
| POW 23-24 | 23.5 | 56.8 | 0.60 | 1.88 | 149 | 12.1 | 45.1 | 21.5 | 15.0 |
| POW 24-25 | 24.5 | 54.6 | 0.59 | 2.06 | 149 | 13.8 | 51.4 | 25.0 | 17.2 |
| POW 25-26 | 25.5 | 52.5 | 0.58 | 2.15 | 155 | 12.7 | 48.9 | 24.0 | 16.3 |
| POW 26-27 | 26.5 | 42.4 | 0.50 | 1.84 | 147 | 12.0 | 45.8 | 21.9 | 15.1 |
| POW 27-28 | 27.5 | 44.5 | 0.52 | 1.80 | 125 | 14.2 | 52.8 | 22.7 | 16.3 |
| POW 28-29 | 28.5 | 58.8 | 0.71 | 1.80 | 128 | 17.8 | 65.6 | 27.1 | 19.4 |
| POW 29-30 | 29.5 | 52.6 | 0.72 | 1.65 | 122 | 17.2 | 63.9 | 25.4 | 18.6 |

^A^ mg HC/g – milligrams of hydrocarbon per gram of sediment; ^B^ *IS –* insufficient sample.

***Table ST3:*** *Concentrations of As, S, Fe, Mn and organic matter fractions (S1, S2, S3, TOC) from the Bulldog Lake (BUL) sediment core (64.03785° N, 111.18337° W)*

| **Sample** | **Depth** | **As** | **S** | **Fe** | **Mn** | **S1** | **S2** | **S3** | **TOC** |
| --- | --- | --- | --- | --- | --- | --- | --- | --- | --- |
| *Units* | *cm* | *mg·kg^-1^* | *wt. %* | *wt. %* | *mg·kg^-1^* | *mg HC/g^A^* | *mg HC/g^A^* | *mg HC/g^A^* | *wt. %* |
| *Detection Limit* | *--* | *0.1* | *0.02* | *0.01* | *1.0* | *0.01* | *0.01* | *0.01* | *--* |
| BUL 0-1 | 0.5 | 590 | 0.46 | 4.09 | 993 | 12.3 | 35.9 | 23.8 | 13.3 |
| BUL 1-2 | 1.5 | 351 | 0.83 | 2.94 | 609 | 10.8 | 29.3 | 16.1 | 10.2 |
| BUL 2-3 | 2.5 | 1,010 | 1.02 | 2.86 | 462 | 11.1 | 30.6 | 17.1 | 11.0 |
| BUL 3-4 | 3.5 | 805 | 0.72 | 2.47 | 449 | 12.5 | 33.8 | 17.2 | 11.6 |
| BUL 4-5 | 4.5 | 566 | 0.58 | 2.15 | 442 | 12.0 | 35.1 | 17.0 | 11.8 |
| BUL 5-6 | 5.5 | 305 | 0.44 | 1.92 | 412 | 13.2 | 35.7 | 16.7 | 11.8 |
| BUL 6-7 | 6.5 | 198 | 0.35 | 1.80 | 406 | 12.5 | 36.4 | 16.3 | 11.6 |
| BUL 7-8 | 7.5 | 114 | 0.30 | 1.51 | 381 | 12.6 | 35.9 | 15.7 | 11.4 |
| BUL 8-9 | 8.5 | 103 | 0.27 | 1.47 | 343 | 11.2 | 32.4 | 15.0 | 10.5 |
| BUL 9-10 | 9.5 | 90.4 | 0.27 | 1.54 | 346 | 10.8 | 32.8 | 14.8 | 10.5 |
| BUL 10-11 | 10.5 | 87.9 | 0.27 | 1.58 | 363 | 10.6 | 34.7 | 15.5 | 11.1 |
| BUL 11-12 | 11.5 | 83.5 | 0.28 | 1.63 | 357 | 11.7 | 34.7 | 16.0 | 11.4 |
| BUL 12-13 | 12.5 | 74.5 | 0.26 | 1.70 | 317 | 10.5 | 31.2 | 14.7 | 10.3 |
| BUL 13-14 | 13.5 | 71.2 | 0.23 | 1.80 | 306 | 9.80 | 28.1 | 13.7 | 9.34 |
| BUL 14-15 | 14.5 | 62.7 | 0.23 | 1.71 | 279 | 9.42 | 27.4 | 13.2 | 9.06 |
| BUL 15-16 | 15.5 | 55.8 | 0.21 | 1.69 | 286 | 8.54 | 25.9 | 11.9 | 8.48 |
| BUL 16-17 | 16.5 | 57.6 | 0.22 | 1.69 | 300 | 9.10 | 26.6 | 12.5 | 8.70 |
| BUL 17-18 | 17.5 | 49.7 | 0.21 | 1.70 | 254 | 8.85 | 25.4 | 12.2 | 8.36 |
| BUL 18-19 | 18.5 | 46.5 | 0.19 | 1.69 | 253 | 7.39 | 21.7 | 10.4 | 7.17 |
| BUL 19-20 | 19.5 | 41.4 | 0.14 | 1.67 | 247 | 7.82 | 23.1 | 10.5 | 7.36 |
| BUL 20-21 | 20.5 | 34.2 | 0.15 | 1.57 | 207 | 7.67 | 23.4 | 10.6 | 7.44 |
| BUL 21-22 | 21.5 | 34.4 | 0.15 | 1.55 | 216 | 7.78 | 23.3 | 10.5 | 7.36 |
| BUL 22-23 | 22.5 | 34.3 | 0.18 | 1.53 | 212 | 8.41 | 27.0 | 11.8 | 8.43 |
| BUL 23-24 | 23.5 | 36.2 | 0.19 | 1.5 | 221 | 8.60 | 26.9 | 11.6 | 8.36 |
| BUL 24-25 | 24.5 | 33.1 | 0.19 | 1.42 | 212 | 9.39 | 28.5 | 12.6 | 8.97 |
| BUL 25-26 | 25.5 | 33.0 | 0.19 | 1.35 | 196 | 10.1 | 29.4 | 12.8 | 9.19 |
| BUL 26-27 | 26.5 | 36.4 | 0.22 | 1.47 | 218 | 10.4 | 29.9 | 13.2 | 9.46 |
| BUL 27-28 | 27.5 | 34.5 | 0.22 | 1.46 | 219 | 10.5 | 30.9 | 13.5 | 9.68 |
| BUL 28-29 | 28.5 | 33.1 | 0.21 | 1.39 | 209 | 9.78 | 30.0 | 12.9 | 9.23 |
| BUL 29-30 | 29.5 | 33.0 | 0.20 | 1.36 | 201 | 10.6 | 30.6 | 13.3 | 9.56 |
| BUL 30-31 | 30.5 | 36.4 | 0.20 | 1.40 | 200 | 10.5 | 30.6 | 13.1 | 9.49 |
| BUL 31-32 | 31.5 | 34.5 | 0.23 | 1.50 | 197 | 11.0 | 31.2 | 13.6 | 9.70 |
| BUL 32-33 | 32.5 | 33.1 | 0.23 | 1.45 | 216 | 10.6 | 30.7 | 13.3 | 9.53 |
| BUL 33-34 | 33.5 | 35.6 | 0.23 | 1.38 | 213 | 11.6 | 34.1 | 14.7 | 10.6 |
| BUL 34-35 | 34.5 | 34.2 | 0.20 | 1.37 | 195 | 9.68 | 28.6 | 12.4 | 8.87 |
| BUL 35-36 | 35.5 | 36.8 | 0.19 | 1.41 | 188 | 8.99 | 26.7 | 11.5 | 8.26 |
| BUL 36-37 | 36.5 | 37.5 | 0.19 | 1.31 | 175 | 9.09 | 27.3 | 11.5 | 8.35 |

^A^ mg HC/g – milligrams of hydrocarbon per gram of sediment.

**Table ST4:** Concentrations of As, S, Fe, Mn and organic matter fractions (S1, S2, S3, TOC) from Hambone Lake sediment grab samples.

| **Sample** | **Units** | **Detection Limit** | **HAM1** | | **HAM2** | | **HAM3** | | **HAM4** | **HAM5** | **HAM6** |
| --- | --- | --- | --- | --- | --- | --- | --- | --- | --- | --- | --- |
| Distance from discharge* | *m* | -- | 540 | 515 | | 310 | | 230 | | 125 | 250 |
| Sediment sample depth | *cm* | -- | 0-15 | 0-15 | | 0-15 | | 0-15 | | 0-15 | 0-15 |
| As | *mg·kg^-1^* | *0.01* | 622 | 576 | | 304 | | 195 | | 303 | 80 |
| S | *wt. %* | *0.02* | 1.2 | 1.8 | | 0.99 | | 0.97 | | 1.02 | 0.21 |
| Fe | *wt. %* | *0.01* | 1.59 | 1.80 | | 1.32 | | 1.34 | | 1.20 | 0.84 |
| S1 | *mg HC/g^A^* | *0.01* | 16.7 | 19.6 | | 11.9 | | 11.4 | | 13.6 | 5.48 |
| S2 | *mg HC/g^A^* | *0.01* | 50.8 | | 52.7 | | 45.4 | | 53.4 | 59.7 | 17.3 |
| S3 | *mg HC/g^A^* | *0.01* | 21.4 | | 25.8 | | 16.2 | | 19.0 | 19.9 | 6.49 |
| TOC | *wt. %* | *--* | 14.5 | | 16.0 | | 11.8 | | 14.0 | 15.1 | 4.73 |

* Location shown on Figure 1; ^A^ mg HC/g – milligrams of hydrocarbon per gram of sediment.

**Table ST5:** Arsenic speciation (HG-AFS) and concentrations of dissolved As, S, Fe, Mn in porewaters extracted from the Powder Mag Lake (POW) sediment core (64.05114° N, 111.15042° W).

| **Sample** | **Depth** | **pH** | **S^A^** | **Fe^A^** | **Mn^B^** | **As_tot_ ^C^** | **As (III)^C^** | **As (V)^C^** | **As_R_ ^D^** |
| --- | --- | --- | --- | --- | --- | --- | --- | --- | --- |
| *Units* | *cm* | *s.u.* | *mg·L^-1^* | *mg·L^-1^* | *mg·kg^-1^* | *µg·L^-1^* | *µg·L^-1^* | *µg·L^-1^* | *µg·L^-1^* |
| *Detection Limit* | *--* |  | *0.05* | *0.005* | *0.1* | *0.09* | *0.09* | *0.09* | *--* |
| POW 0-1 | 0.5 | 7.4 | 71.3 | 2.18 | 372 | 383 | 80.0 | 284 | 18.6 |
| POW 1-2 | 1.5 | 7.0 | 66.1 | 0.90 | 460 | 304 | 53.5 | 189 | 60.9 |
| POW 2-3 | 2.5 | 7.5 | 72.6 | 0.30 | 463 | 58.9 | 7.30 | 41.1 | 10.4 |
| POW 3-4 | 3.5 | 7.4 | 73.2 | 1.04 | 507 | 28.8 | 7.50 | 13.4 | 8.00 |
| POW 4-5 | 4.5 | 7.4 | 77.5 | 0.38 | 381 | 89.0 | 30.3 | 49.7 | 9.00 |
| POW 5-6 | 5.5 | 7.0 | 83.2 | 0.09 | 345 | 57.3 | 14.4 | 37.9 | 5.00 |
| POW 6-7 | 6.5 | 7.2 | 88.7 | 0.03 | 335 | 16.0 | 2.60 | 10.0 | 3.50 |
| POW 7-8 | 7.5 | 7.1 | 94.0 | 1.21 | 413 | 10.4 | 2.10 | 6.20 | 2.10 |
| POW 8-9 | 8.5 | 6.4 | 99.4 | 0.04 | 267 | 8.70 | 2.50 | 4.40 | 1.90 |
| POW 9-10 | 9.5 | 6.3 | 106 | 0.02 | 323 | 4.20 | 1.00 | 1.70 | 1.50 |
| POW 10-11 | 10.5 | 6.5 | 111 | 1.65 | 478 | 2.30 | 0.40 | 0.70 | 1.20 |
| POW 11-12 | 11.5 | 6.7 | 112 | 3.76 | 544 | 2.40 | 0.40 | 1.00 | 0.90 |
| POW 12-13 | 12.5 | 6.7 | 114 | 0.06 | 524 | 1.80 | 0.40 | 0.40 | 0.90 |
| POW 13-14 | 13.5 | 6.7 | 117 | 1.42 | 518 | 1.80 | 0.30 | 0.30 | 1.20 |
| POW 14-15 | 14.5 | 6.7 | 118 | 0.87 | 558 | 1.30 | 0.20 | 0.20 | 0.90 |
| POW 15-16 | 15.5 | 6.4 | 119 | 0.86 | 525 | 1.30 | 0.20 | 0.20 | 0.90 |
| POW 16-17 | 16.5 | 6.5 | 116 | 20.3 | 819 | 1.50 | 0.30 | 0.40 | 0.80 |
| POW 17-18 | 17.5 | 6.5 | 117 | 1.63 | 585 | 0.80 | 0.30 | 0.10 | 0.40 |
| POW 18-19 | 18.5 | 6.2 | 116 | 8.16 | 723 | 0.50 | 0.20 | 0.20 | 0.10 |
| POW 19-20 | 19.5 | 6.3 | 116 | 1.87 | 639 | 2.40 | 0.90 | 0.30 | 1.20 |
| POW 20-21 | 20.5 | 6.2 | 113 | 0.17 | 566 | 1.80 | 1.70 | 0.10 | 0.00 |
| POW 21-22 | 21.5 | 6.3 | 109 | 30.8 | 915 | 0.60 | 0.30 | 0.10 | 0.20 |
| POW 22-23 | 22.5 | 6.1 | 102 | 6.27 | 818 | 3.40 | 1.60 | 0.30 | 1.50 |
| POW 23-24 | 23.5 | 6.7 | 99.4 | 13.8 | 825 | 1.60 | 0.40 | 0.20 | 0.90 |
| POW 24-25 | 24.5 | 6.1 | 94.0 | 2.04 | 655 | 5.20 | 3.10 | 0.30 | 1.70 |
| POW 25-26 | 25.5 | 6.3 | 92.1 | 24.8 | 843 | 2.20 | 0.90 | 0.40 | 0.90 |
| POW 26-27 | 26.5 | 6.0 | 87.8 | 18.4 | 810 | 1.40 | 0.70 | 0.20 | 0.50 |
| POW 27-28 | 27.5 | 6.4 | 83.6 | 32.9 | 823 | 2.70 | 0.90 | 0.30 | 1.50 |
| POW 28-29 | 28.5 | 6.2 | 78.3 | 26.7 | 767 | 4.70 | 0.80 | 3.00 | 0.80 |
| POW 29-30 | 29.5 | 5.9 | 71.9 | 5.09 | 629 | 3.20 | 2.10 | 0.30 | 0.90 |

^A^ ICP-OES; ^B^ ICP-MS; ^C^ HG-AFS; ^D^ Calculated from HG-AFS results

**Table ST6:** Arsenic speciation (HG-AFS) and concentrations of dissolved As, S, Fe, Mn in porewaters extracted from the Bulldog Lake (BUL) sediment core (64.03785° N, 111.18337° W)

| **Sample** | **Depth** | **pH** | **S^A^** | **Fe^A^** | **Mn^B^** | **As_tot_ ^C^** | **As (III)^C^** | **As (V)^C^** | **As_R_ ^D^** |
| --- | --- | --- | --- | --- | --- | --- | --- | --- | --- |
| *Units* | *cm* | *s.u.* | *mg·L^-1^* | *mg·L^-1^* | *mg·kg^-1^* | *µg·L^-1^* | *µg·L^-1^* | *µg·L^-1^* | *µg·L^-1^* |
| *Detection Limit* | *--* |  | *0.05* | *0.005* | *0.1* | *0.09* | *0.09* | *0.09* | *--* |
| BUL 0-1 | 0.5 | 5.7 | 0.22 | 5.02 | 2756 | 74.3 | 32.84 | 27.9 | 13.5 |
| BUL 1-2 | 1.5 | 5.8 | 1.77 | 0.22 | 1646 | 67.7 | 11.35 | 30.7 | 25.6 |
| BUL 2-3 | 2.5 | 5.7 | 2.87 | 1.82 | 1473 | 82.7 | 12.42 | 67.7 | 2.61 |
| BUL 3-4 | 3.5 | 5.9 | 5.52 | 0.02 | 1309 | 21.4 | 3.77 | 9.92 | 7.73 |
| BUL 4-5 | 4.5 | 5.7 | 10.8 | 0.02 | 932 | 33.2 | 2.46 | 20.0 | 10.8 |
| BUL 5-6 | 5.5 | 5.7 | 10.0 | 0.11 | 797 | 22.2 | 2.06 | 17.1 | 3.04 |
| BUL 6-7 | 6.5 | 5.8 | 6.54 | 0.18 | 34.8 | 37.6 | 3.58 | 28.8 | 5.25 |
| BUL 7-8 | 7.5 | 5.7 | 2.32 | 0.12 | 610 | 30.5 | 2.68 | 24.6 | 3.18 |
| BUL 8-9 | 8.5 | 5.6 | 4.34 | 0.16 | 28.0 | 24.3 | 2.06 | 20.5 | 1.72 |
| BUL 9-10 | 9.5 | 5.6 | 1.75 | 0.13 | 213 | 20.5 | 1.79 | 13.2 | 5.55 |
| BUL 10-11 | 10.5 | 5.6 | 5.19 | 0.18 | 78.8 | 20.4 | 1.99 | 17.6 | 0.75 |
| BUL 11-12 | 11.5 | 5.7 | 1.96 | 0.14 | 223 | 16.8 | 2.24 | 14.2 | 0.30 |
| BUL 12-13 | 12.5 | 5.8 | 2.50 | 0.13 | 66.7 | 16.8 | 1.30 | 11.6 | 3.84 |
| BUL 13-14 | 13.5 | 5.9 | 2.40 | 0.15 | 25.5 | 17.2 | 1.50 | 14.4 | 1.27 |
| BUL 14-15 | 14.5 | 5.7 | 1.45 | 0.08 | 378 | 9.42 | 1.08 | 6.25 | 2.09 |
| BUL 15-16 | 15.5 | 5.8 | 3.37 | 0.13 | 53.7 | 12.1 | 1.23 | 10.1 | 0.80 |
| BUL 16-17 | 16.5 | 5.6 | 2.01 | 0.04 | 65.2 | 11.8 | 1.93 | 8.71 | 1.18 |
| BUL 17-18 | 17.5 | 5.6 | 3.86 | 0.02 | 24.5 | 12.2 | 1.12 | 8.33 | 2.72 |
| BUL 18-19 | 18.5 | 5.7 | 2.03 | 0.02 | 66.7 | 10.4 | 1.15 | 6.45 | 2.75 |
| BUL 19-20 | 19.5 | 5.7 | 1.26 | 0.10 | 18.1 | 11.6 | 3.73 | 6.85 | 1.06 |
| BUL 20-21 | 20.5 | 5.7 | 0.40 | 0.01 | 198 | 12.2 | 4.92 | 5.24 | 2.07 |
| BUL 21-22 | 21.5 | 5.8 | 0.59 | 0.07 | 27.5 | 13.2 | 5.18 | 5.67 | 2.34 |
| BUL 22-23 | 22.5 | 5.7 | 0.45 | 0.03 | 46.9 | 16.1 | 11.14 | 2.27 | 2.69 |
| BUL 23-24 | 23.5 | 5.7 | 1.52 | 0.02 | 43.8 | 12.8 | 2.62 | 7.68 | 2.53 |
| BUL 24-25 | 24.5 | 5.7 | 1.03 | 0.02 | 74.4 | 12.0 | 3.21 | 4.08 | 4.73 |
| BUL 25-26 | 25.5 | 5.7 | 0.55 | 0.03 | 175 | 17.3 | 10.80 | 2.25 | 4.27 |
| BUL 26-27 | 26.5 | 5.7 | 0.80 | 0.01 | 139 | 16.9 | 3.09 | 11.3 | 2.52 |
| BUL 27-28 | 27.5 | 5.7 | 0.80 | 0.01 | 67.4 | 13.2 | 5.55 | 3.71 | 3.89 |
| BUL 28-29 | 28.5 | 5.8 | 0.63 | 0.01 | 187 | 15.4 | 8.77 | 2.21 | 4.40 |
| BUL 29-30 | 29.5 | -- | 0.22 | -- | -- | -- | -- | -- | -- |
| BUL 31-32 | 31.5 | 5.7 | 1.77 | 0.03 | 50.4 | 16.0 | 3.28 | 8.95 | 3.73 |
| BUL 32-33 | 32.5 | 5.7 | 2.87 | 0.09 | 36.4 | 15.2 | 6.08 | 7.53 | 1.55 |
| BUL 33-34 | 33.5 | 5.9 | 5.52 | 0.04 | 17.4 | 19.2 | 11.76 | 6.33 | 1.06 |
| BUL 34-35 | 34.5 | 5.7 | 10.6 | 0.01 | 265 | 13.2 | 13.07 | <0.09 | -- |
| BUL 35-36 | 35.5 | 5.8 | 10.0 | 0.02 | 63.0 | 19.9 | 10.46 | 3.94 | 5.45 |
| BUL 36-37 | 36.5 | 5.7 | 6.54 | < 0.005 | 230 | 14.4 | 11.63 | 0.32 | 2.43 |

^A^ ICP-OES; ^B^ ICP-MS; ^C^ HG-AFS; ^D^ Calculated from HG-AFS results

***Table ST7:*** *Solid-phase arsenic speciation in selected lake sediments based on* *linear combination fitting of XANES spectra. Spectra and results of LCF*

| Depth | As(-I)-S | As(III)-S^a^ | As(III)-O^a^ | As(V)-O^a^ | Fitted Sum^b^ | R value^c^ | Reduced χ^2^ |
| --- | --- | --- | --- | --- | --- | --- | --- |
| *Reference* | *Arsenopyrite* | *Orpiment* | *Hematite-As(III)* | *HFO-As(V)* | *--* | *--* | *--* |
| *cm* | *%* | *%* | *%* | *%* | *%* | *--* | *--* |
| Powder Mag Lake | | | | | | | |
| 1 - 2 | -- | 28.0 | 25.0 | 47.0 | 101.6 | 0.00281 | 0.00092 |
| 3 - 4 | 16.0 | 41.0 | 24.0 | 19.0 | 101.1 | 0.00234 | 0.00068 |
| 6 - 7 | -- | 37.0 | 29.0 | 34.0 | 100.9 | 0.00372 | 0.00115 |
| Bulldog Lake | | | | | | | |
| 0 – 1 | -- | 5.00 | 40.0 | 55.0 | 100.6 | 0.00119 | 0.00043 |
| 1 – 2 | 22.0 | 55.0 | 14.0 | 9.00 | 101.0 | 0.00346 | 0.00102 |
| 2 – 3 | 14.0 | 60.0 | 17.0 | 10.0 | 101.0 | 0.00545 | 0.00170 |
| 5 – 6 | -- | 44.0 | 26.0 | 30.0 | 101.3 | 0.00347 | 0.00107 |
| Hambone Lake | | | | | | | |
| HAM1 | 56.0 | 10.0 | 26.0 | 8.00 | 100.0 | 0.01035 | 0.00236 |
| HAM2 | 63.0 | -- | 28.0 | 9.00 | 98.2 | 0.00936 | 0.00193 |
| HAM5 | 28.0 | 25.0 | 21.0 | 25.0 | 100.5 | 0.00681 | 0.00170 |

^a^ Component sums were normalized to 100%; ^b^ Fitted sum of all references before normalization to a sum of 100%; ^c^ Mean square misfit between the data and the fit.

***Figure SF1****:* XANES analysis of merged selected sediment samples and results of linear combination fitting (best fit and the residual) for Bulldog Lake, Powder Mag Lake, and Hambone Lake.

*
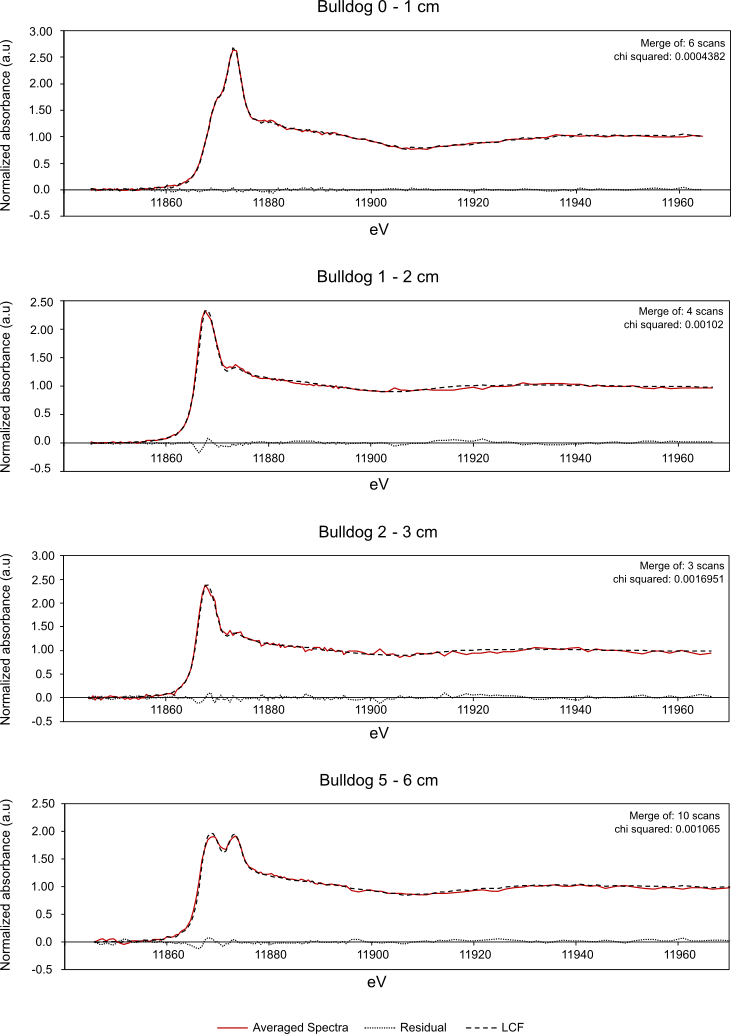
*


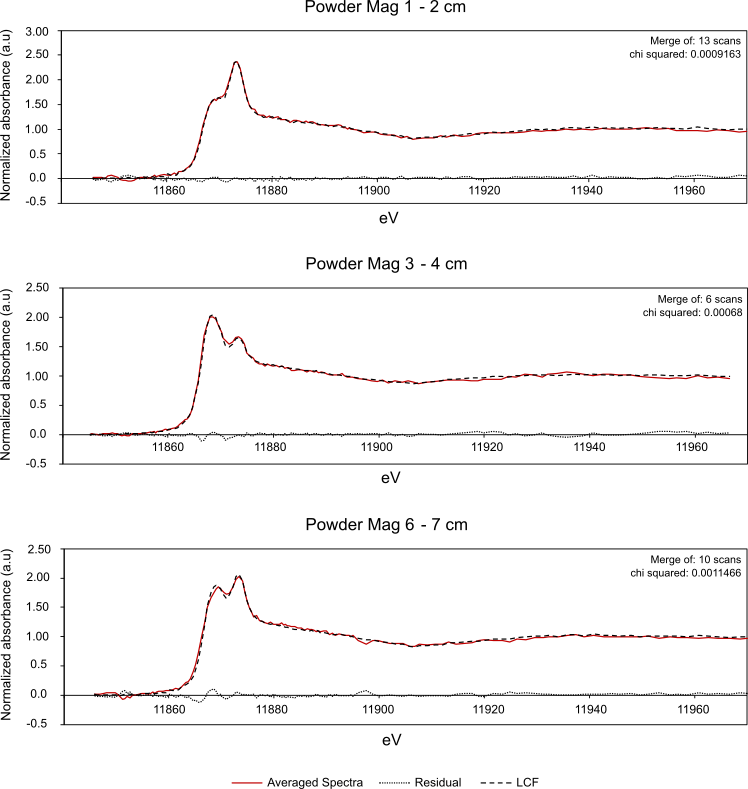


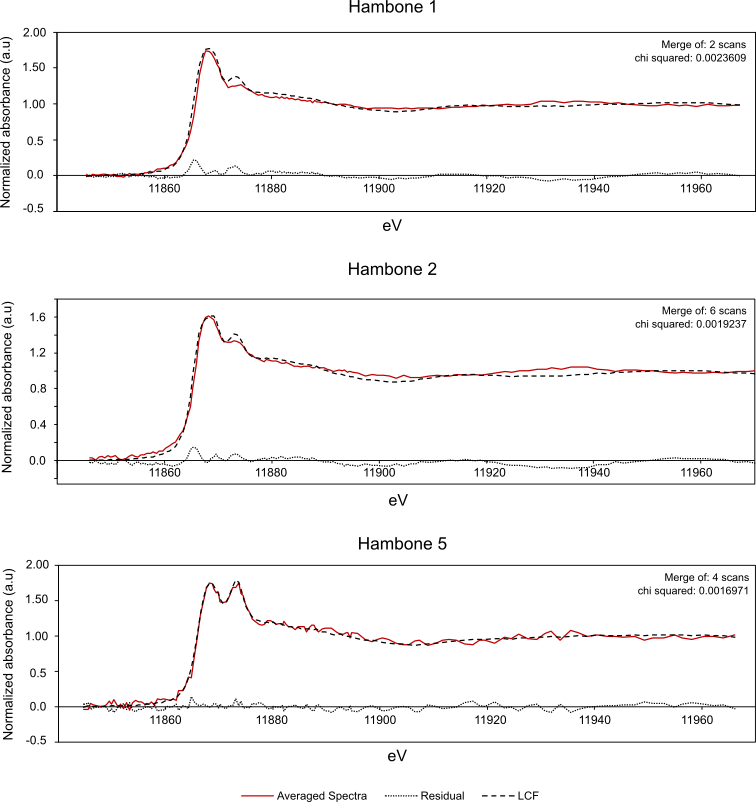


***Figure SF2****: Additional µXRF maps with corresponding µXRD spectra from Bulldog (a, b, c) and Hambone (d, e) lakes; (a) Qtz – quartz, Lep – lepidocrocite, Mgh – maghemite; (b) Gt – goethite, Mkw – mackinawite; (c) Tnt – titanite, Fh – ferrihydrite, Gt – goethite; (d) Gt – goethite; (e) Mkw – mackiniwite, Mgh – maghemite.*


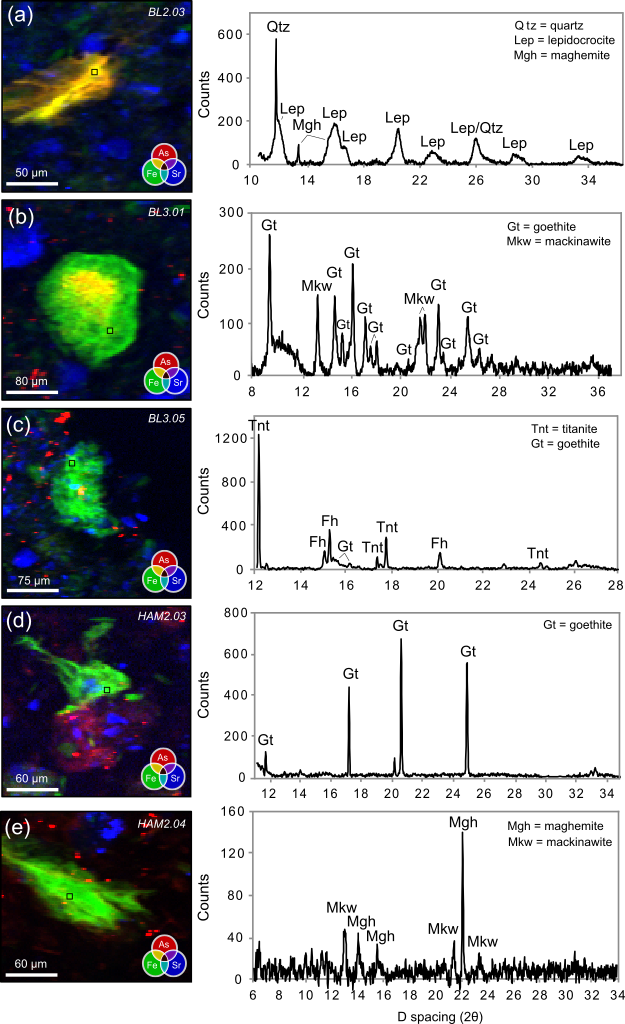

Supplement: Supplementary file 1 — Supplementary file1 (DOCX 618 kb) [file 12665_2022_10213_MOESM1_ESM.docx]
